# Mechanical Joining of Fibre Reinforced Polymer Composites to Metals—A Review. Part II: Riveting, Clinching, Non-Adhesive Form-Locked Joints, Pin and Loop Joining

**DOI:** 10.3390/polym12081681

**Published:** 2020-07-28

**Authors:** Anna Galińska, Cezary Galiński

**Affiliations:** Institute of Aeronautics and Applied Mechanics, Faculty of Power and Aeronautical Engineering, Warsaw University of Technology, Nowowiejska 24, 00-665 Warsaw, Poland; cegal@meil.pw.edu.pl

**Keywords:** fiber reinforced polymer composite, joining, riveting, clinching, strength

## Abstract

As fiber reinforced plastic composites gain an increasingly larger share in aerospace structures, the problem of joining them with metal elements becomes significant. The current paper is the second part of the literature review, which gathers and evaluates knowledge about methods suitable for the mechanical joining of composite and metal elements. This paper reviews the joining methods other than bolted joining, which are discussed in the first part of the review, namely self-piercing riveting, friction riveting, clinching, non-adhesive form-locked joints, pin joints, and loop joints. Some of those methods are full-fledged and employed in commercial applications, whereas others are merely ideas tested at the level of specimens. The current review describes the ideas and the qualities of the joining methods as well as the experimental work carried out so far. The summary section of this paper contains a comparison of those methods with the reference to their qualities, which is important from the point of view of a composite structure designer: possibility of the joint disassembly, damages induced in composite, complication level, weight penalty, range of possible materials to be joined, and the joint strength.

## 1. Introduction

Fibre reinforced composite materials continue to gain progressively larger share in the structures of modern aircraft. The amounts of composite laminates used in the Boeing 787 Dreamliner and Airbus A350 exceed 50% of the vehicle weight [1,2]. Since the metal elements are and will be indispensable in aerospace structures, the problem of joining composite and metal elements becomes more and more important as the share of composite elements in the structures increases. The joint used in a composite structure is usually the weakest point of the structure, and thus determines the structural efficiency [3]. A separate part of this literature review is devoted to the bolted joining of composite and metal elements [4], as it is the most widely used method of mechanical joining. However, the bolted joining of composite materials has several drawbacks, resulting mainly from the need of drilling holes. The process of drilling disrupts the continuity of the fibres reducing the load carrying capacity [5,6,7] and causes damages (such as delamination, fibre pull-out and microbuckling [7]). Therefore, the other methods of mechanical joining have been developed and some of them were specially dedicated for joining composite to metals. Those methods include self-piercing riveting, friction riveting, clinching, non-adhesive form-locked joints, pin joints, and loop joints. Some of those methods do not require making holes in cured composite material (non-adhesive form-locked joints, pin joints and loop joints) and thus eliminate the disruption of the fibre continuity and drilling-induced damages, whereas, the others as riveting and clinching disrupt the structure of the composite significantly. Some of the presented methods, including non-adhesive form-locked joints, pin joints, and loop joints, take the advantage of the fibrous nature of the composites in order to transfer loads onto the metal structure in a sophisticated yet clever way. Some of the methods, as self-piercing riveting or form-locked joints, have been successfully developed and employed in commercial applications, whereas, the others, like pin and loop joints, are merely ideas, tested only at the specimen level. Some of the methods present the possibility of joint disassembly without destroying it, whereas some are permanent. Some methods can be used to join any composite and some of them are limited only to the composites with thermoplastic matrix.

The number, diversity, and complexity of mechanical methods of joining composite and metal materials makes the choice of the proper method tedious and difficult. Furthermore, the recent review work on joining FRP composites to metal is moderate. Amancio-Filho and dos Santos described trends in joining of polymers and polymer-metal hybrid structures including bolted joining, clinching, welding and friction riveting [8]. Kah et al. reviewed techniques for joining metals and polymers in 2014 [9]. Those methods included adhesive bonding, bolted joining, and welding. In the most comprehensive work, Pramanik et al. presented a review of joining methods of carbon fibre reinforced polymer composites and aluminium alloys, including adhesive bonding, loop joining, self-piercing riveting, bolted joining, clinching and welding [10]. Dawei et al. provided the most recent works reviewing several mechanical and non-mechanical joining methods, namely: adhesive bonding, bolted joining, pin joining, welding, self-piercing riveting, and clinching [11,12]. Since none of those works either focus solely on the mechanical joining methods or cover all the developed methods of mechanical joining, and some of the joining methods have been omitted entirely, the current work presents a review which covers all the mechanical joining methods. The current paper along with its first part [4] provides a literature review which describes the level of development, and the advantages and disadvantages of mechanical methods of joining composite and metal materials. The current paper omits bolted joining, which is reviewed thoroughly in the first part of this review [4]. The subsequent sections of the current work describe two methods of riveting used in composite materials: self-piercing riveting and friction riveting, method of joining by mechanical clinching, non-adhesive form-locked joints designed in order to introduce a concentrated force into a composite structure, pin joints which join the composite to metal by arrays of pins protruding from metal elements inserted into composite during curing process and loop joints which consist in entangling reinforcing fibres into loops protruding from metal elements. As those methods vary significantly, the summary section of this paper contains a comparison of those methods with reference to their features, which is important from the point of view of a composite structure designer: possibility of the joint disassembly, damages induced in composite, complication level, weight penalty, range of possible materials to be joined, and the joint strength. This section is divided into subsections which facilitate fast familiarization with the most important conclusions.

## 2. Riveted Joints 

Conventional riveting does not apply to composite materials because loads experienced during the riveting process destroy the composite matrix around the rivet [13], therefore this method cannot be used to make responsible connections of metal and composite parts. However, recently, two methods of riveting have been developed, which do not require drilling holes in composite materials and impose smaller mechanical assembly loads. Those methods are called self-piercing riveting (SPR) and friction riveting.

### 2.1. Self-Piercing Riveting

**Figure 1:**
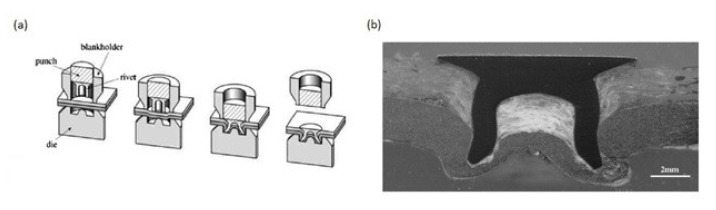
(**a**) Self-piercing riveting process, (**b**) cross-section of two dissimilar materials joined by self-piercing riveting [16].

The process of self-piercing riveting consists in joining two stacked sheets of the same or dissimilar materials by a rivet which is forced into the stack by a punch. The rivet pierces the top material through and partially pierces into the bottom material. The die on the bottom side of the stacked sheets causes the rivet to flare under the force, creating a mechanical interlock [14,15]. Both the rivet and the bottom plate are subjected to plastic deformations [16]. The self-piercing riveting process is presented in Figure 1a and the cross-section of two dissimilar materials joined by a self-piercing rivet is presented in Figure 1b [16].

This process has gained wide diffusion in the automotive industry, due to the increasing use of materials alternative to steel that are difficult or impossible to join using traditional techniques [17,18]. Recently, it has been used for joining FRP to metal sheets. The advantages of the method are following:

- The process does not require pre-drilled holes, and thus it is carried out in one operation [14,15,19], which saves production time and costs [16,20]. However, it is also possible to drill or cut a hole in the upper plate before the riveting.
- No surface pre-treatment is needed [20,21].
- The process is operator-friendly, and it emits only low noise, no fumes [20], and is easy to automate [19].

**Figure 2:**
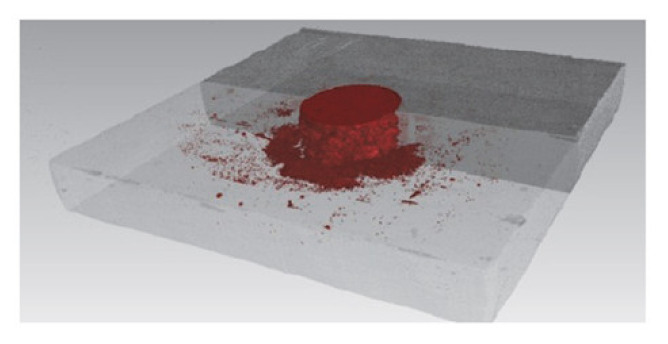
Volumetric computed tomography scan of delaminations around a punched hole in carbon fibre reinforced plastic during SPR [29].

One disadvantage of the process is the fact that both sides of the joint must be accessible [16], which limits the use of the technique only to the part of the structures. However, this disadvantage is well recognized and quite common in joining techniques (appears, e.g., also in bolted joining). Another disadvantage is that it is impossible to disassembly the joint without destroying it. Also galvanic corrosion may occur in this joining method if rivets made of aluminium alloys or steel are used along with CFRP composite [22,23] due to the difference in the potential conductivity of the composite and the aforementioned metals [24,25]. Usually, the fasteners are required to be made of stainless steel or even titanium to minimize the corrosion [26]. However, the most serious and not fully recognized drawback of the technique seems to be the problem of process-induced damages in joining fibre-reinforced composite materials. From this point of view the SPR method is not appropriate for brittle materials [27,28,29]. Unlike most metals, composite materials rarely exhibit plastic behaviour. Therefore, as the punch energy cannot be utilized by plastic deformation, it may cause matrix and fibre damages, delaminations, and cracking in the composite material [26,29]. These damages may propagate when the joint is loaded under fatigue cyclic stresses and eventually cause premature joint failure by the composite rupture [21,30]. Moreover, the fibres are cut by the rivet, which causes the disruption of the load paths, as in the case of hole drilling. The mechanism causing delamination is also similar as in the case of drilling. As the rivet is punched through the subsequent plies, the thickness and the resulting stiffness of the intact plies is decreasing. Thus, the last plies are subjected to severe bending which may lead to a delamination if the peeling stress of the plies is exceeded [29]. Figure 2 shows delaminations around a punched hole in carbon fibre reinforced plastic revealed by a tomography scan [29]. 

**Figure 3:**
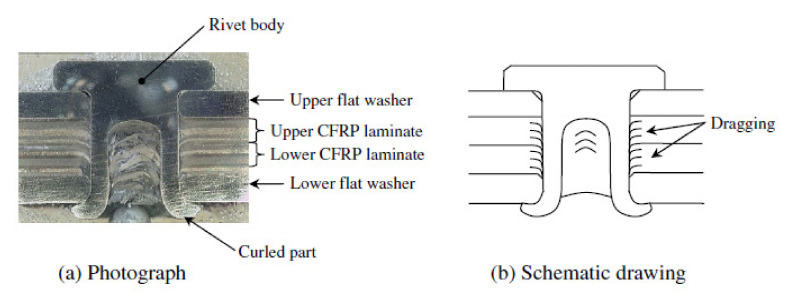
Cross-section of CFRP laminate joined by SPR (**a**) photograph, (**b**) schematic drawing [21].

Ueda et al. found, on the basis of the cross-sections of SPR joined composite materials, that dragging of composite plies due to the punching appears around the hole (Figure 3) [21]. Delamination was not apparent on visual inspection because the delaminated plies were clamped together by the rivet [21].

Zhang and Yang compared joining of polyamide 6 matrix thermoplastic composite reinforced with glass and carbon short fibers with aluminium substrate by SPR method [31]. The joints were made without pre-drilling holes in the upper substrate. The joining was performed in four configurations: GFRP composite on the top and aluminium on the bottom (GFRP/Al), aluminium on the top and GFRP composite on the bottom (Al/GFRP), CFRP composite on the top and aluminium on the bottom (CFRP/Al) and aluminium on the top and CFRP composite on the bottom (Al/GFRP). It was found out that the GFRP composite can be placed either on the top or on the bottom of the joint, the joint obtained was always acceptable. For CFRP, acceptable joints were obtained only when the composite panel was placed on the bottom. This was attributed to the fact that carbon fibers have lower ductility than glass fibers, so they crack more easily during SPR operation which causes large shear deformations of the upper substrate [31]. It was also noticed that some cracks also appear when the aluminium sheet is placed on the top in CFRP composite. The strengths of the joints were obtained by SLS tests. The higher strength was achieved for GFRP/Al joints (3.75 kN) and similar strengths were achieved for Al/GFRP and Al/CFRP joints (2.6 kN and 2.4 kN respectively) [31]. However, the strength of CFRP/Al joints was virtually non-existent [31] which corresponds well with the observations of cracking in the specimens. Settineri et al. presented a research, in which the influence of various geometrical parameters of rivets and dies on SPR joints was investigated [17]. Combinations of six different materials, including metals, polymers, and composites, were tested in a single lap shear test. The highest shear strength was achieved for the combination of a low carbon steel and a thermoplastic produced from polybutylene terephthalate (PBT) and polycarbonate (PC) joined by a rivet with the diameter of 3.9 mm with the use of a die with the diameter of 8 mm and depth 1.75 mm. It was found that the process depends strongly on the geometrical parameters of the rivet, on the riveting force and on the metallic material [17]. Di Franco et al. investigated the carbon-epoxy laminate with stacking sequence 0°/45° riveted by SPR to aluminium alloy blanks [14]. The single lap shear tests were conducted. It was observed that the fundamental failure mechanism in SPR joined aluminium and carbon reinforced composite panel is bearing [14]. Gay et al. tested the fatigue strength of glass fibre reinforced polyamide composite joined by SPR to aluminium alloy sheet [20,30]. Two types of rivets were used: with domed (protruding) head and with counter-sunk head. The rivet shanks have diameters of 5 mm. The joint with the domed headed rivet achieved higher fatigue strength than the joint with the countersunk rivet. For instance, the fatigue limit strength at 2 × 10^6^ cycles is higher by 22% for the domed headed rivets than for the countersunk rivets [20]. It can be expected, that the mechanism which deteriorates the performance of the countersunk rivet is similar as in the case of countersunk bolts [32]. However, this was not investigated further. The temperature has also determining influence on the fatigue strength of the SPR composite joints. The glass/polyamide–aluminium specimen had 30% better performance in 23 °C than in 40 °C temperature, which was attributed to the polyamide resin sensitivity to the temperature [20]. Di Franco et al. investigated joining of carbon fibre reinforced laminate and aluminium alloy element by SPR joining and adhesive bonding by epoxy resin [16]. Single lap joints with two rivets placed longitudinally were tested. Three distances between the rivets (30, 45, and 60 mm) were investigated in static and fatigue tests. The mechanical connections were made with the use of rivets with flat countersunk heads. The rivets used were made of austenitic steel and were designed to join materials of total thickness equal to 4.5 mm. The joints with the distance between the two rivets equal to 60 mm showed the best static behaviour in terms of tensile strength [16]. Ueda et al. proposed a self-piercing riveting process, which was modified by adding two washers in order to suppress the delamination appearing during the riveting. Single lap joints of a carbon–epoxy composite were tested [21]. The experimental results showed that the tensile strength of a modified SPR joint was slightly higher than that of a bolted joint. The tension–tension fatigue tests have shown that the fatigue limit at N = 10^7^ cycles was about 50% of the tensile joint strength [21]. Di Franco et al. investigated the fatigue behavior of aluminium/CFRP joints obtained by SPR method [18] in SLS tests. The fatigue tests were preceded by static tests which allowed to choose optimal riveting conditions. In the fatigue tests cracking of the composite panel around the rivet was observed for a fatigue life under 200,000 for large values of fatigue load (3200 N). Net section cracking of the aluminium sheet was found out as lower values of fatigue loads were applied (2400 N) [18]. The failure of the riveted joint during the fatigue tests was localized either in the aluminium blank or in the composite panel, while during the static tests, the failure occurred always in the composite panel [18]. However, no thorough explanation of why the failure modes changed was provided. The comparison of mechanical behavior of three types of joints: hybrid SPR-bonded, simple SPR and simple bonded joints was made by di Franco et al. [19]. The joints of aluminium and CFRP were investigated in single lap shear tests. The hybrid joints achieved the strength of approx. 5800 N, the bonded, of 5000 N, and the SPR, of approx. 3700 N [19]. It was concluded that the addition of the SPR rivet to the adhesive bond increases the strength of the joint, because the rivet yields compression force on the bond which stops the adhesive failure and prevents the joint failure after the adhesive failed completely [19]. However, though the hybrid SPR-bonded method seems to be indeed a promising enhancement of the SPR method, it was not explained which mechanism caused the increase of the SPR-bonded joints in respect of the simple SPR joints. 

General characteristics of self-piercing riveting are presented in Table 1. Applicable standards are:

- DVS-EFB 3440-2:2006-07
- DVS-EFB 3490:2015-10

### 2.2. Friction Riveting

**Figure 4:**
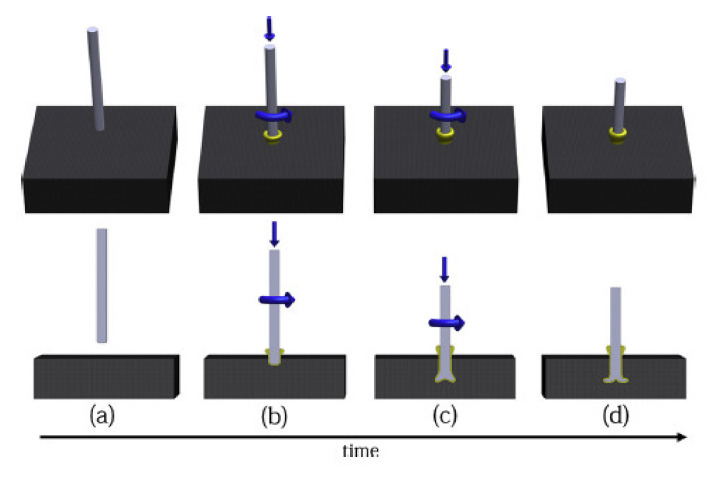
Friction riveting process steps: (**a**) positioning of the joined parts, (**b**) friction heat generates a molten layer of polymer and the rivet penetrates it, (**c**) increasing the axial force in order to widen and anchor river tip, (**d**) consolidation of the joint [36].

Friction riveting was first developed to create joints to metals in thermoplastic materials [33], but recently several works considered the use of this method for joining fibre reinforced polymer composites. Further use of this joining method may include a wide range of practical applications. So far, Blaga et al. proposed and evaluated the feasibility of using this method for the construction of composite emergency bridges [34,35]. Friction riveting consists in anchoring a metallic rivet inside a polymer or polymer composite base plate [36]. Firstly, a cylindrical rivet is placed in the spindle of the riveting equipment, and a plate made of thermoplastic polymer or thermoplastic matrix composite is fixed beneath it (Figure 4a). The process comprises of three steps: the friction step, the forging step, and the consolidation [37]. The rotating rivet is moved toward the plate (Figure 4b). When it touches the plate, the combination of rotation and axial pressure generates frictional heat, which melts the polymer around the rivet tip. The rivet is continuously fed through the plate. The heat generation rate during this phase increases, and the heat input grows to exceed the heat outflow due to the low thermal conductivity of the polymer [34,36]. Due to the local increase in temperature, the rivet tip becomes plasticised (Figure 4c) [36]. At this point, the rotation is decelerated and the rivet tip is suppressed by the additional axial pressure. This forming step widens the rivet tip, creating a mushroom-like shape. During the last step the joint consolidates and cools under the constant external pressure, while the rivet remains strongly anchored in the polymeric plate, forming a metallic-insert joint (Figure 4d) [36].

The friction riveting method has the following advantages:

- No surface preparation is needed [34].
- The process does not need pre-drilled holes in composite material, which reduces the time and number of assembly steps [34,35].
- Hermetically sealed joints can be created [35].
- In addition, it has the following disadvantages:
- Only thermoplastic matrix composites can be joined by friction riveting, since this method of joining requires polymer matrix to melt [36], which is impossible in the case of thermosetting polymers.
- There is no possibility of disassembling the joint without destroying it [38].
- Only spot-like joints can be obtained [38].
- As the process requires heat generation, there is the possibility of thermal degradation of the polymer matrix due to the low thermal conductivity of both reinforcing fibre and polymeric matrix [39,40,41].
- Galvanic corrosion may occur in this joining method if rivets made of aluminium alloys or steel are used along with CFRP composite [22,23].

**Figure 5:**
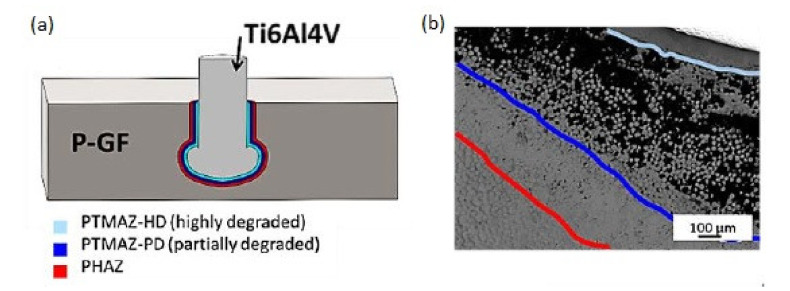
(**a**) Schematic drawing of the zones affected by heat and deformation, (**b**) microstructure of polymer heat affected zone PHAZ (red line) which includes: partially degraded polymer thermo-mechanically affected zone PTMAZ-PD (navy blue) and highly degraded polymer thermo-mechanically affected zone PTMAZ-HD (light blue) [41].

Considering that the thermal degradation is directly coupled with the mechanical performance of polymers, this should be avoided or minimised in friction riveting in order to guarantee the joint mechanical integrity [40]. Amancio-Filho investigated the thermal degradation of polyetherimide joined by friction riveting [40]. Joints were produced by keeping setup joining time and joining pressure constant at 3 s and 1.1 MPa (11 bar), respectively, while rotation speed varied within the range 15,000–21,000 rpm [40]. In order to evaluate the temperature profile during the joining process, two joints per condition were analysed by infrared thermography [40]. Due to the low thermal conductivity of the polymer, it is assumed that the polymer softened through frictional heating and pressed off as flash will not have enough time to cool down before it leaves the polymer base plate [40]. In other words, the average temperature measured in the softened flash is considered nearly the same as in the molten layer inside the polymer plate [40]. The expelled material was measured to have temperature 350–475 °C, which is within the initial range of thermal degradation for the studied polymer [40]. Consequently, thermally degraded material will be present inside the joint. Temperature and heating rates were found to increase with the rotational speed, while the heating time (in this case assumed to be the thermal degradation dwell time) increased only slightly [40]. Borba et al. also conducted a research in which the thermal degradation of the polymer during friction riveting was investigated [41]. The tested composite was glass fibre-polyester. Different values of rotational speed and friction time were used. During the joining process, the increase in temperature was monitored on the polymeric flash expelled from the composite plate during the joining process using an infrared thermo-camera system. For some of the welding configurations the peak temperatures measured during welding were well above the degradation temperature for polyester matrix (761 °C for rotational speed 1000 rpm and friction time 1.2 s) [41]. It should be noted that such a high temperature would be destructive to any available polymer matrix, let alone polyester. The microscopic examination of the joints cross-sections showed, however, that the zone affected by the thermal degradation is relatively small (Figure 5) [41].

**Figure 6:**
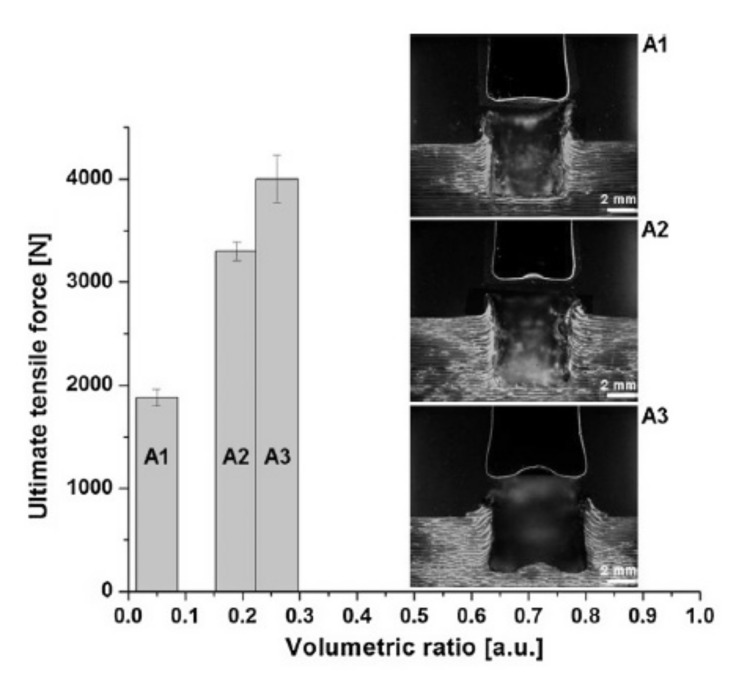
Ultimate tensile forces for each specimen riveted with different rotational speeds 8000 rpm (Specimen A1), 9000 rpm (Specimen A2) and 10,000 rpm (Specimen A3) [34].

Metal/composite specimens joined by friction riveting were also tested in tensile single lap shear test [41]. The highest strength (6.7 ± 1.6 kN) was obtained for intermediate riveting conditions (rotational speed 900 rpm and friction time 1.2 s), while for higher rotational speed (rotational speed 1000 rpm and friction time 1.2 s), the mechanical performance (5.3 ± 1.3 kN) was probably negatively influenced by excessive polymer degradation, leading to a decrease of about 28% of the ultimate lap shear force [41]. Specimens failed mainly by two combinations of failure modes. The strongest joints for each set of investigated conditions failed by the combination of bearing in the composite and metallic plates and shear through the metallic rivet, whereas, the others failed by the combination of bearing and pull-out of the rivet [41]. Blaga et al. investigated the friction riveting of polyetherimide glass fibre reinforced laminates [34,35]. The titanium rivets with a 5-mm diameter were used [34]. The influence of the rotational speed on the joint formation and mechanical performance was evaluated under the joining conditions: constant joining time (3.2 s), constant joining pressure (1.0 MPa) and varying rotational speeds of: 8000 rpm (Specimen A1), 9000 rpm (Specimen A2) and 10,000 rpm (Specimen A3) [34]. The joints were then tested in pull-out tests. Figure 6 shows the ultimate tensile forces for each specimen riveted with a different rotational speed. The highest strength was achieved for 10,000 rpm speed. The anchoring efficiency of the rivet can be estimated through the volumetric ratio (VR), which takes into account the ratio of the volume of dislocated polymeric material, the volume of the deformed metallic rivet and the volume of the remaining polymeric material over the deformed shape of the rivet [34]. The strength was related to the volumetric ratio, leading to the conclusion that a higher rotational speed causes a higher volumetric ratio, which in turn increases the pull-out strength. 

**Figure 7:**
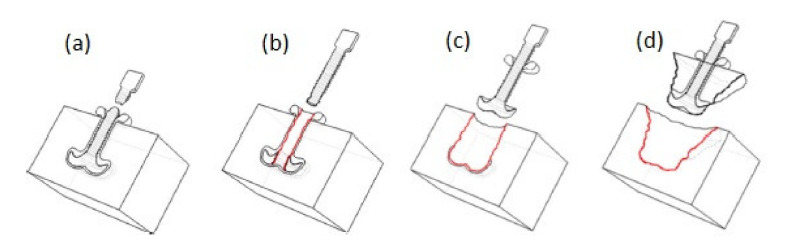
Failure mechanisms of friction-riveted joints: (**a**) rivet failure, (**b**) rivet pull-out with backward plunge, (**c**) full rivet pull-out, (**d**) rivet pull-out [42].

The measured process temperatures, influenced mainly by the rotational speed of the process, ranged between 430 °C and 464 °C [34]. The monitored process temperatures can be related to the small level of thermal degradation in the polymeric matrix and to the formation of the rivet anchoring zone [34]. In their second work Blaga et al. considered riveting of the same material (glass fibre reinforced polyetherimide) by threaded titanium rivets (M5-thread rivets, pitch of diameter 4.6 mm, with a length of 60 mm) [35]. The GF-PEI plates were riveted to aluminium plates, perforated with a hole of 5 mm diameter, and subjected to single lap shear tension test. The influence of the following parameters on the lap shear strength was studied: rotational speed (RS): 8000, 10,000, 12,000 rpm, friction time (FT): 700, 1200, 1500 ms, forging time (FOT): 1200, 1850, 2500 ms, forging pressure (FOP): 0.6, 0.7, 0.8 MPa. The highest strength (199 MPa) was achieved for RS = 12,000 rpm, FT = 700 ms, FOT = 2500 ns and FOP = 0.7 MPa. Some specimens failed by bearing mode, some by bolt shearing [35]. Generally, it can be stated that the joining time (JT) represented by the components FT and FOT and the rotational speed (RS) are the parameters with the largest influence on the lap-shear strength in the studied material combination [35]. The mechanical performance of friction-riveted joints was also compared with bolted joints [35]. Bolted lap-shear specimens were produced analogically to the friction-riveted lap-shear specimens, with identical materials and specimen geometries, and tested according to the same standards. The ultimate lap shear strength of the bolted joined specimens was by 12.8% lower than the strength of the best series of the friction riveted joints. However, as all the bolted specimens failed by the bolt shearing, the strength of these specimens was underestimated. The experiment, in which both riveted and bolted specimens would fail by bearing should be conducted in order to compare the strengths of those two joining methods in an unbiased way. The temperature during riveting was measured by an infrared camera [35]. As in the case of the other works [40,41], the temperature was recorded during joining from the expelled polymeric matrix material, on the contact area between the rivet and polymer [35]. The process temperature varied between 450 °C and 600 °C. No significant thermal degradation of PEI is expected due to its high thermal resistance [35] because this polymer displays accelerated susceptibility to thermal degradation only at temperatures above 600 °C and long exposure times [40], which are not expected to occur in the scope of the discussed work [35]. Altmeyer et al. investigated the feasibility of friction riveting on short carbon fibre-reinforced polyether ether ketone (PEEK) [36,37]. The impact of rotational speed, friction time, friction pressure and forging pressure on the joint formation and its performance was evaluated by pull-out test. The rivets with a 5-mm diameter were made of titanium. During this study, the rotational speed (RS) varied from 18,000 rpm to 20,000 rpm, the friction pressure (FP) varied from 0.7 MPa to 0.9 MPa, the forging pressure (FoP) varied from 0.9 MPa to 1 MPa, the friction time (FT) varied from 1 to 1.5 s, and the forging time (FoT) was kept constant (10 s) to ensure sufficient and uniform joint consolidation [36]. The highest pull-out strength (10.7 kN) was achieved for the three sets of parameters: RS = 18,000 rpm, FT = 1.5 ms, FP = 0.7 MPa, FoP = 1 MPa; RS = 20,000 rpm, FT = 1 ms, FP = 0.9 MPa, FoP = 1 MPa and RS = 20,000 rpm, FT = 1.5 ms, FP = 0.7 MPa, FoP = 1 MPa. The higher the RS, FT and FoP are, the higher the pull-out force is. The increases of these parameters lead to the higher mechanical energy input values, leaving additional heat available for deforming and widening the rivet tip [36]. The wider the rivet tip is, the larger the volume of polymer is above the deformed rivet tip. Therefore, higher forces are required to pull the metallic insert out of the polymeric substrate [36]. The tendency of the rivet tip widening was described by a parameter called ‘mushrooming efficiency’ [37]. It combines the initial rivet diameter (D) and the width of the deformed rivet tip (w) into a factor that gives the widening of the rivet tip as a percentage of the undeformed diameter of the rivet [37]. It was used to estimate the anchoring efficiency based on the area of contact between the rivet anchoring zone and the composite. The mushrooming efficiency ranges from 34.4% to 91.4% [37]. The possible failure modes of friction-riveted joints in a pull-out test are presented in Figure 7 [42].

**Figure 8:**
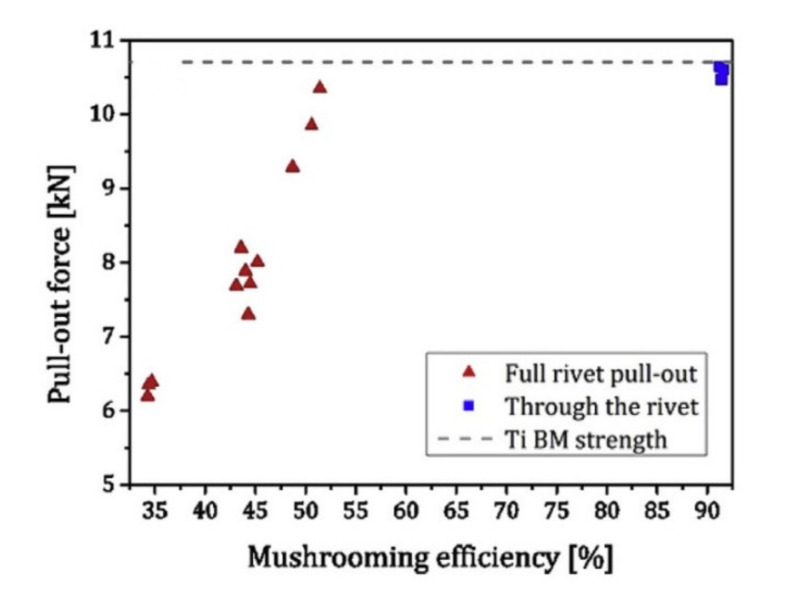
Influence of mushrooming efficiency on pull-out force [37].

A mushrooming efficiency of 70% leads to the maximum performance of a metallic-insert joint [36]. At this threshold, the failure mode changes from failure mode pull-out of rivet (Figure 7c) to failure mode rivet failure (Figure 7a). The influence of the mushrooming efficiency on the pull-out force and the mode of failure is presented in Figure 8 [37].

As in the rivet failure mode the rivet fails before the joint, the strength 10.7 kN should be considered as the highest strength for friction joining in this configuration. Any further increase in the pull-out force can be achieved only by using a different rivet material [37].

Min et al. investigated the process window for FSBR (friction stir blind riveting) joining of CFRP composite to CFPR composite or to aluminium alloy [43]. Both combinations, CFRP substrate on top of the aluminium one (CFRP-AA6111 joints) and reversed (AA6111-CFRP joints), were tested. The best results were achieved for the first combination (CFRP-AA6111 joints). The brittleness of the CFRP material was found to be the deterministic factor that limits the size of the process window. In each material combination, a quality issues, i.e., delamination on the backside of the CFRP sheet, were observed at relatively low spindle speeds and higher feed rates [43]. Without any support below the bottom surface of the CFRP bottom sheet, the CFRP-CFRP and the AA6111-CFRP joints could not be produced without quality issues under the same process settings that produced good CFRP-AA6111 joints [43]. Table 2 presents the obtained process windows for AA6111-CFRP and CFRP-AA6111 joints.

After the quality assessment, the joints were subjected to single lap joint testing. It was discovered that the strength of the joints is only dependent on the material stack-up and insensitive to the process parameters that fall within the process window. The quality issue associated with the brittle spalling off the bottom surface of CFRP leads only to a 10% reduction in strength in CFRP-CFRP and AA6111-CFRP joints and does not affect the strength of CFRP-AA6111 joints [43]. Based on these observations, it can be stated that the FSBR process is robust in producing consistent joint strength. Similar method is used also to join thin aluminium plates [44,45].

General characteristics of friction riveting are presented in Table 3. 

## 3. Mechanical Clinching

**Figure 9:**
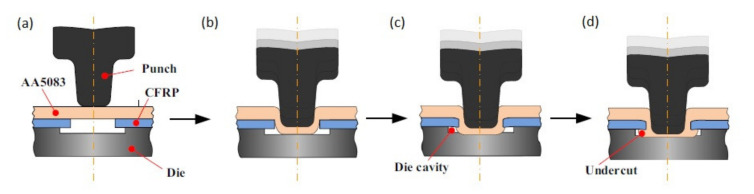
Clinching process: (**a**) positioning of the joined sheets, (**b**) straining of the upper sheet, (**c**) spreading of the upper sheet in the die in order to create an interlocking joint, (**d**) clinched joint [46].

Clinching is a method of joining carbon fibre reinforced plastics (CFRP) and ductile materials [46]. This method was originally developed to join metal sheets including high strength steels and aluminium alloys [47]. It has been used in automotive [48]. Then, it has been extended to join many other materials including joining fibre reinforced composites to metal alloys [47]. Generally clinching consists in plasticising of either composite or metal sheet in order to build geometrical interlocking, however, it is preferable to place the material with higher ductility (metal) on the punch side, because during clinching the punch-side sheet undergoes severe plastic deformation [49]. The most common way to perform clinching of composite and metal consists in forcing the metal component into the composite component in order to produce mechanical interlocking (Figure 9). The composite component is placed on a die and covered with the metal component (Figure 9a). The punch is positioned over the die cavity and it forces the metal component into the cavity (Figure 9b). The composite component can have a predrilled hole necessary to assure interlocking as it is presented in Figure 9 [46,48,50] or the hole can be punched during the clinching process [47,49,51]. The pre-cutting of the hole reduces the material damages compared to punching the hole in composite [51], however, it also adds additional step to the joining process. The punch is forced further in order to push the metal sheet to fit the die (Figure 9c) in order to create interlocking joint with undercuts (Figure 9d) [46].

Clinching process has the following advantages:

- No additional elements are needed. Therefore, it is a fast, low cost, and lightweight process [47,49,52,53]
- Predrilled holes or surface preparation are not necessary in the case of thermoplastic matrices which simplifies the process [51,53]

**Figure 10:**
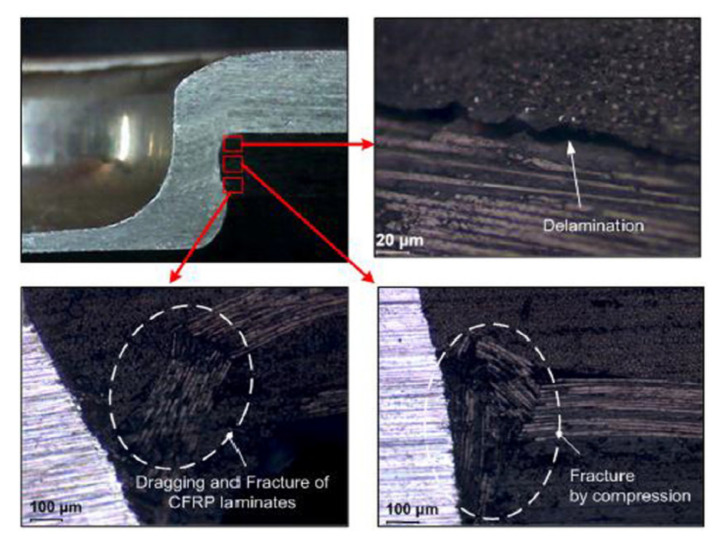
Damages of composite materials visible in cross-section of hole-clinched joint [46].

However, it has also disadvantages:

- Once the joint is clinched it is impossible to disassembly it without destroying components [53]
- As the process consists in the plastic deformation the joined materials have to exhibit certain ductility, so that not all materials can be subjected to clinching [46,49]. It is preferable to place the material with higher ductility on the punch side, because, during clinching, the punch side sheet undergoes severe plastic deformation [49,51].
- The clinching process can cause some material damage in the composite materials (dragging, delaminations and cracking) (Figure 10) [46,54], especially if the hole is not pre-cut, but punched.
- Galvanic corrosion may occur in this joining method if a substrate made of aluminium alloys or steel is used along with a CFRP composite [22,23].

Lee et al. conducted single lap shear tests of AA6061 alloy and the CFRP sheets joined by clinching [54]. The specimens had different composite sheet thickness: 1.0, 1.2, 1.4 or 1.6 mm. The experiments have shown that the failure mode changes with the composite substrate thickness. For 1.0- and 1.2-mm thicknesses the specimens failed by the button separation (pull-out of the metal inclusion from the composite), but for 1.4- and 1.6-mm thicknesses, the failure mode changed to the neck fracture. The failure loads were higher for the higher thicknesses (2.75 kN and 3.34 kN respectively) compared to the lower thicknesses (failure loads 2.0–2.5 kN) [54]. Lee et al. also investigated experimentally the effect of the tool shape on the hole clinching [52]. The hole clinching process of joining CFRP laminates with aluminium alloy and high strength steel sheets was studied. It was concluded that [52]:

- The punch diameter has a significant influence on the joint quality: the neck-thickness is decreased and the undercut is increased with the increase of the punch diameter.
- The neck formation is also severely influenced by the punch corner radius. Sharp punch corners induce the concentration of deformation at the neck of the upper sheet, which decreases the neck-thickness, so the punches with tight radii are suitable for punching materials which have sufficient ductility, such as SPRC440. Blunt punch corners are suitable for more fragile materials, such as Al6061, and reduce the damage at the neck, but on the other hand, expand the hole in the lower sheet.
- The die depth also influences the quality of the joints. When the die is too shallow the undercut creation process is disturbed. On the other hand, too deep a die may cause the fracture of the joint neck in the joining process.

**Figure 11:**
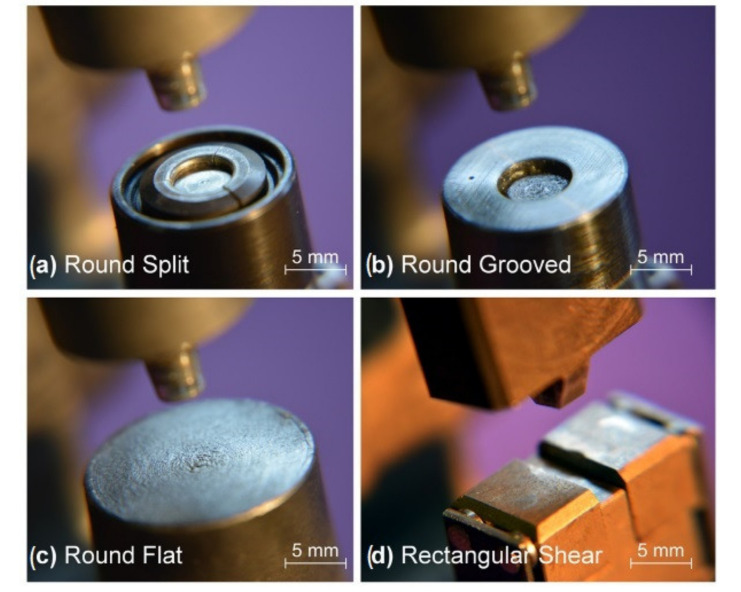
Clinching dies: (**a**) round split; (**b**) round grooved; (**c**) round flat and (**d**) rectangular [49].

The best failure strength of single lap shear (SLS) specimens (2.69 kN) was achieved for hole clinching process of CFRP laminates and Al6061 with flat die and with sharp punch corner [52]. The joining of CFRP (carbon fabric preimpreganted with epoxy resin) laminates and SPRC440 steel by the hole-clinching process was also investigated in another work [48]. Holes of 8.2 mm in diameter were drilled in the composite and the steel was punched into them. In the experiments corner radius of 0.5 mm and three punch diameters, 6.6 mm, 6.8 mm, and 7.0 mm, were used. Single lap shear tests of the specimens were performed. For the joints clinched with the punch diameters 6.6mm and 6.8 mm the average maximum loads were 2.91 kN and 3.36 kN and the failure mode was button separation (pull-out) [48]. For the punch diameter of 7.0 mm, the maximum load dropped to 2.25 kN and the failure mode changed to neck fracture [48]. The failure load increased when the punch diameter was increased from 6.6mm to 6.8 mm, because it caused the increase of the undercut and hole expansion [48]. However, with the further increase of the punch diameter to 7.0 mm the failure load decreased, because the neck became so thin, that it was fractured before the button was pulled out from the composite [48]. Lambiase and Ko also compared the results of carbon-epoxy composite and aluminium alloy clinching with different punch diameters [47]. Four punches used in this work had three different diameters (3, 4 or 4.5 mm) and two taper angles (6° or 12°). The holes in the composite materials were not pre-cut, but punched during the clinching process. The results of the strengths of single lap shear specimens clinched with these punches were investigated. During the single lap shear tests, all the examined joints failed by pull-out followed by bearing of CFRP, regardless of the type of punch used [47]. It was concluded that the punch diameter has significant influence on the joint quality: with the increase of the diameter the undercut is increased, but larger punch diameter induces also more material damage in the CFRP composite. The increase in the taper angle causes greater damage on the CFRP sheet, which is due to the delay in the fibre cut along the developing hole owing to higher hydrostatic stress produced during the offsetting phase [47]. Reduction of the taper angle from 12° to 6° allowed an increase in the shear strength of 50% [47]. Lambiase et al. also investigated the influence of the die shape on the clinched joint quality [49]. Different clinching tools were adopted to join aluminium sheets with GFRP sheets by mechanical clinching, namely: a round split (extensible) die, a round grooved die, a round flat die and a rectangular tool (Figure 11) [49].

The clinching process was performed without pre-cutting holes in the composite sheet. Single lap shear tests of the specimens were carried out. In the case of the grooved and split dies part of the composite was stuck in the grooves and the splits proving very difficult to be removed, which makes those dies unsuitable for the commercial manufacturing process [49]. On the other hand, flat dies generally promote small undercuts [49]. Split dies allow to produce joints of better quality [49]. In order to prevent the entrapment of the composite material within the splits, a loose sheet of aluminium was placed between the composite and the split die, which successfully prevented this undesirable phenomenon. Single lap shear tests confirmed that the use of the split die yields better results than the use of the flat die. Joints produced with the split die had higher strength than the joints produced with the flat dies (1.9 kN vs. 1.3 and 2.1 kN vs. 0.8 kN) [49]. The clinched joints failed by pull-out or neck fracture (or combination of both), depending on their geometry features (neck thickness and undercut) [49]. Lee et al. proposed a new type of hole clinching tool called a spring die [46]. This tool was designed to improve the hole clinching process of CFRP and aluminium alloy (AA5083). In the spring die two pads supported by a coil spring are employed to improve the formability of ductile materials and to reduce damages to CFRP laminates by increasing the compressive hydrostatic stress during the hole clinching process. The microscopic observations of the clinched joint cross-sections have shown that the spring die is suitable to prevent the fracture and delamination of CFRP laminates, which are main defects in hole clinching process [46]. Moreover, it was observed that the spring die can prevent the damage accumulation at the neck of the upper sheet by the means of compressive hydrostatic stress [46].

An investigation which analyses two-stepped clinching based on reshaping deformation that follows mechanical clinching, as a method to improve the mechanical behaviour of clinched connections performed on hybrid metal-composites joints was presented by Lambiase and Ko [51]. The clinching of AA6024 aluminium alloy and carbon fibre reinforced thermosetting composite was performed. Single lap shear tests were performed in order to verify the effectiveness of the reshaping method. The split die was preferred to a fixed (grooved) one because it generally produces larger undercuts with lower forming forces and enables an easier extraction of composite crumbles from the die cavity [55]. In the second step (reshaping), the height of the punch was varied in order to produce different punch-die cavity geometries [51]. Two values of the reshaping force 20.5 kN and 28.8 kN chosen according to the preliminary test results were used. The reshaping step resulted in appearance of several damages in the composite material: buckling, delaminations and cracking [51]. Those defects resulted in the reduction of the strength of the joint in majority of the reshaped joints. However, under optimal conditions of reshaping (reshaping force 28.8 kN and reshaping depth 0.5 mm) the shear strength was increased by 32% compared to the reference joints. The improvement of the mechanical behaviour is attributed mainly to the increase in undercut [51]. Lambiase and Paoletti employed a rotating tool in order to join thin aluminium sheets of a carbon–epoxy composite by means of clinching [50]. The friction-assisted clinching was used to join aluminium alloy component with CFRP composite component. The process was named friction-assisted clinching. The aim of the tool rotation was to soften the aluminium component by friction generated heat and thus reduce the joining forces. According to the achieved results, the employment of friction clinching allowed to increase dramatically the material formability and enabled the production of joints without fractures even with sharp punch tools [50]. The employment of the rotating tool also enabled a great plunging load reduction. Actually, conventional clinching generally involves forces ranging between 10 kN and 20 kN, while in friction assisted clinching a force of 300 N enabled complete joining [50]. On the other hand, the time of the process increased to 30 s compared to 1 s time used in conventional clinching [50]. The specimens were tested in single lap shear experiment. The specimens failed by either pull-out caused by insufficient undercuts or neck fracture caused by insufficient neck thickness [50]. The clinching process was monitored by an IR camera. Despite high temperatures registered during the process (up to 300 °C), no evident sign of thermal degradation of the epoxy matrix was observed at the CFRP aluminium interface [50]. This was attributed to the low contact pressure exerted by the aluminium on the CFRP side wall during great part of the process duration.

**Figure 12:**
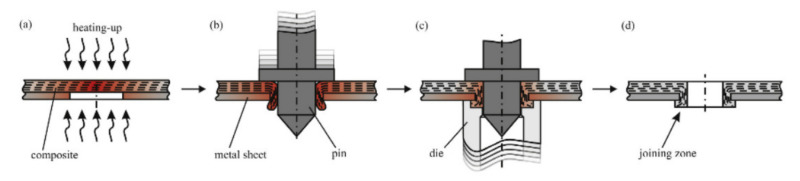
Schematic illustration of the novel thermoclinching process: (**a**) positioning of the joining partners and heating-up of the pre-cut joining zone; (**b**) permeating of the fiber reinforced structure with the tapered pin; (**c**) forming of the undercut with the die and (**d**) demoulding of the thermoclinched joint [53].

Apart from the conventional clinching process in which the metal component is plasticized in order to create lock in the hole made in the composite component, there have been several attempts made in order to design different processes, which can be incorporated to the group of ‘mechanical clinching’ methods. In those processes the composite component is the one which is softened to create a lock in the hole of the metal component. Only thermoplastic matrix composites are suitable to be joined by those processes, because the thermoset matrix, once cured, cannot be plasticized again and would crack under high strains required in clinching. Gude et al. developed a method called thermoclinching in order to join steel sheets with glass fabric reinforced polypropylene [53]. The composite was pre-cut in the joining zone and locally heated to increase its plastic deformation ability, shifted through the pre-punched metal sheet by a tapered pin and compressed from the backside by a die to generate a form-locked joint (Figure 12a–c). After cooling and solidification, the mould was opened to reveal the geometrical joint (Figure 12d) [53].

**Figure 13:**
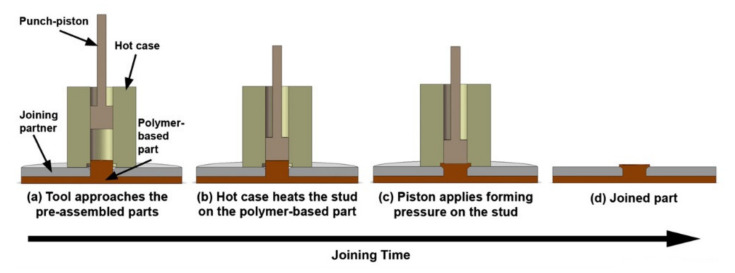
Steps of the ICJ process: (**a**) tool approaches the pre-assembled parts, (**b**) hot case heats the stud on the polymer-based part, (**c**) piston applies forming pressure on the stud, (**d**) joined part [56].

The joints were tested in single lap shear experiments and failed by either pull out (3kN) or shear (2 kN) of the composite formed lock, depending on its geometrical features [53]. Abibe et al. proposed a joining method called injection clinching joining (ICJ) [56,57]. This method starts when a composite part with a protruding stud is pre-assembled with a joining partner that contains a hole, so that the stud fits in the hole. The hot case and punch-piston tool system approaches the pre-assembled parts, with the hot case containing the polymeric stud (Figure 13a) [56]. The stud is heated at a certain processing temperature for a predetermined joining time (Figure 13b), after which the punch-piston pushes the softened polymer into the hole (Figure 13c) [56]. Then, the system is cooled under pressure to reduce the polymer thermal relaxation and the joint is consolidated (Figure 13d). By the end of the process, a joint with no additional parts other than the joining partners and with the polymeric stud working as a rivet to the joint is obtained [56].

**Figure 14:**
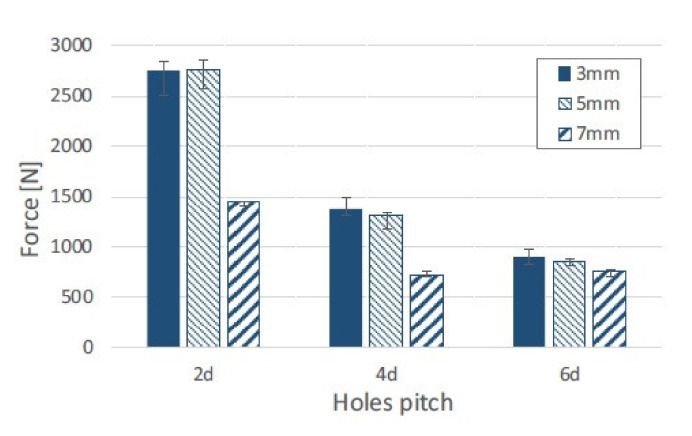
Strength of specimens with different hole diameters (3 mm, 5 mm and 7 mm) and different pitches between the holes (2d, 4d and 6d) [58].

**Figure 15:**
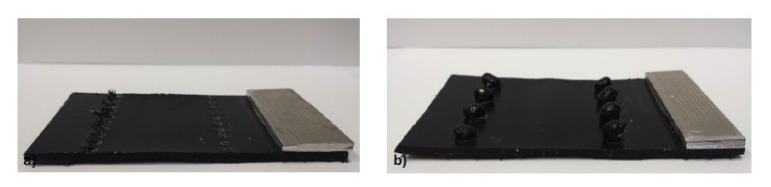
Failure modes in friction stir welded specimens: (**a**) shearing of the protrusions; (**b**) separation of the sheets [58].

Joints of short glass fibre-reinforced polyamide 6,6 and aluminium alloy were produced using the injection clinching joining process [56,57]. The holes in the metal substrate were threaded in order to provide more efficient anchoring of the composite [57]. The micrographic pictures of the joints revealed that the thermal processing in 250 °C can induce void formation, while processing at 300 °C caused voids on the deformed stud and a large loss of polymer mass [57]. Single lap shear tests of the joints were performed. The impact of the joining time, processing temperature and drying treatment on the maximum load of a reference sample was investigated [56]. The reference sample achieved an average maximum load of 942 ± 77 N. Reducing the joining time (from 180 s to 15 s) raised the average value to 1112 ± 102 N, while reducing the processing temperature (from 300 °C to 250 °C) improved the performance to 1807 ± 114 N [56]. Drying of the composite material yielded a positive effect allowing to achieve 1114 ± 79 N for conditioning 24 h at 60 °C and 1208 ± 77 N for conditioning 24 h at 120 °C [56]. The processing temperature appears to have the most prominent effect on the joint performance. Buffa et al. proposed a new friction stir welding approach to join mechanically AA6082-T6 to self-reinforced polypropylene [58]. The aluminum sheet was pre-holed along both sides and placed on the top of the composite sheet. A pinless tool was plunged into the aluminium sheet and moved along the longitudinal direction with constant feed rate. The heat generated by the friction force softened the top sheet and was transferred by thermal conduction to the bottom sheet [58]. Due to the friction heat and the pressure composite extrusions filled the holes in the aluminium sheet. Once cooled down, the extruded polypropylene created a mechanical bond between the aluminium and the composite [58]. The specimens with different hole diameters and different hole pitch were subjected to shear test. The results of the tests are presented in Figure 14. The failed specimens with different failure modes are presented in Figure 15.

It was found that the vertical force on the tool, as well as the tool diameter, rotation, and feed rate, must be carefully selected in order to generate correct heat which does not melt the composite but, at the same time softens it enabling the backward extrusion [58]. 

General characteristics of clinching are presented in Table 4. Applicable standards are:

- DVS-EFB 3420:2012-02
- DVS-EFB 3450-1:2007-05

## 4. Non-Adhesive Form-Locked Joint

**Figure 16:**
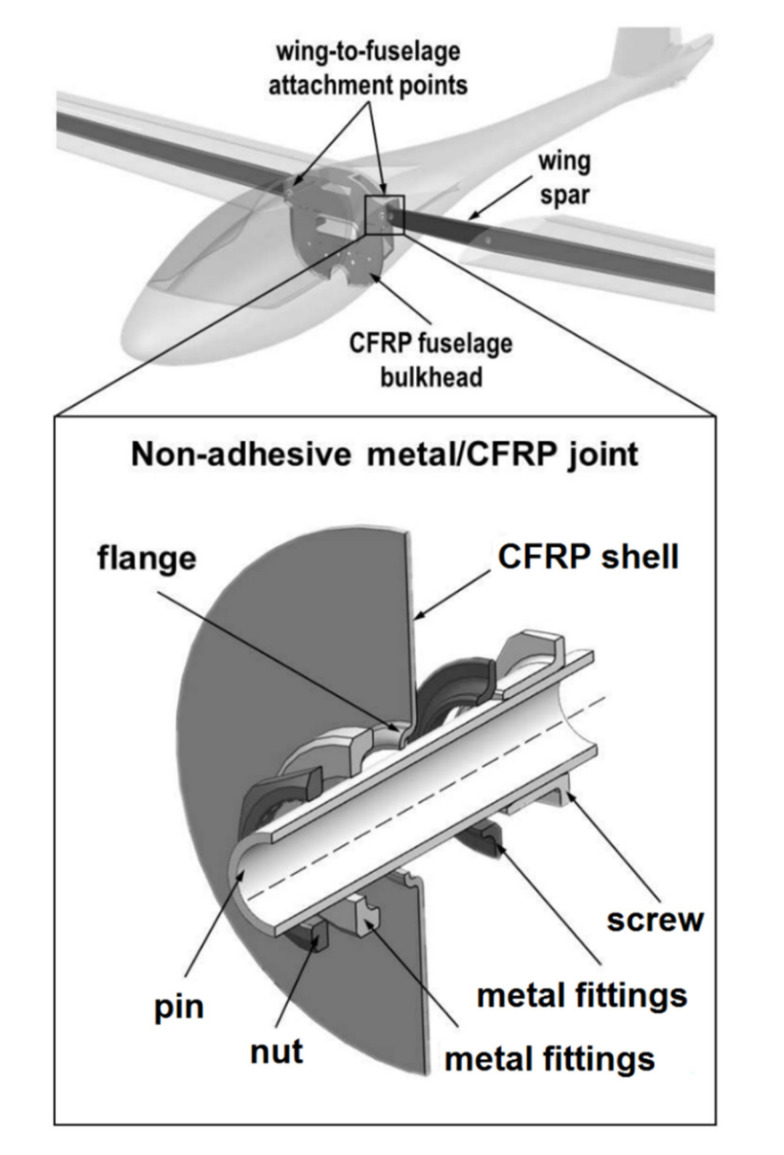
Fuselage-wing connection with the use of non-adhesive form-locked joint of the AOS-71 motoglider [60].

When large elements are assembled often occurs a need for introducing a concentrated force into a composite structure. In such cases a single massive joint which could withstand the force has to be designed. The simplest example of such a joint is a bolted joint. However, phenomena such as delaminations, fibre pull-outs, and microbuckling [7], which occur during drilling the hole for the joint in composite, deteriorate its performance [4]. Moreover, a big hole required for the joint would cause considerable disruption of the load paths by removing significant part of the reinforcing fibres. Those disadvantages prompt engineers to look for better solutions for introducing a concentrated force into a composite structure. An example of such an idea which was developed and successfully used in engineering cases is non-adhesive form-locked joint designed in Warsaw University of Technology [59]. The joints of this type were used in the PW family of commercial and experimental gliders and motogliders (PW-5, PW-6, and AOS-71) in a fuselage–wing connection (Figure 16). 

The non-adhesive form-locked joint is manufactured along with the composite structure. The hole is cut in dry fabric which is then infused by resin or in uncured prepreg. Metal rings are then fitted to the hole so that the edge of the hole is curled up and locked between them. The structure is then cured in a vacuum bag or in an autoclave. After the curing a screw with a hole in it and a nut are fitted into the hole of the joint in order to clench it (Figure 16). A bolt is put through the hole in the screw to assembly the structure. This solution ensures that the fibres of the composite are attached to the joint in all directions and clenched in it, which means that, unlike in a traditional bolted joint, where the force is introduced only in the area of contact between the bolt and the composite [61], the force is distributed in the composite in all directions. The additional advantage of the non-adhesive form-locked joint is the fact that the hole is cut in the fabric before curing, so the delaminations and other damages caused by the hole drilling in the composite can be avoided. Moreover, the joint can be easily assembled and disassembled at will. On the other hand, the disadvantages of the joint include its significant complication level and the mass increased by the presence of additional metal elements such as the holed screw and the nut. The risk of galvanic corrosion is also significant if the elements of the joint placed in carbon fibre reinforced composite are made of steel or aluminium alloys [22,23].

**Figure 17:**
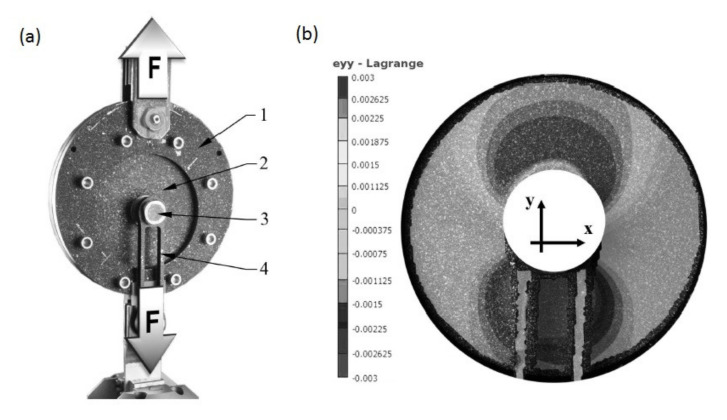
Testing of non-adhesive form-locked joint: (**a**) Experimental set-up: 1—metal grip, 2—laminate plate, 3—bolt, 4—loading bands; (**b**) Experimental (DIC) distribution of strains in θ = 0° direction (loading direction) for force F = 62 kN [60].

An experimental investigation of the strength of the non-adhesive form-locked joint was carried out by Tomasiewicz and Czarnocki [60]. The joints made of stainless steel were manufactured in carbon fabric/epoxy specimens consisting of 16 plies cured OOA. After the curing the nut of the joint was tightened with a torque of 75 Nm [60]. The tensile static tests of two specimens were carried out. A specimen in the testing setup is presented in Figure 17a. The maximum strength obtained by both specimens was 60–70 kN. The strain field over the laminate plate was determined with the use of the 3D digital image correlation (DIC) method [60]. The strains in the load direction are presented in Figure 17b. It can be clearly seen that the concentrated force causes strains in the composite below and above the joint, though the strains below the joint are higher. The significant strains above the joint mean, however, that the stress concentration below the joint is decreased in regard of the equivalent conventional bolted joint.

After the static tests, an inspection of the damage done to the laminate plates was carried out with the help of computed tomography (CT). Several failure modes were distinguished: fiber rupture, matrix cracking, matrix-cracking induced delaminations, and compressive shear [60].

General characteristics of non-adhesive form-locked joining are presented in Table 5.

## 5. Pin Joining

**Figure 18:**
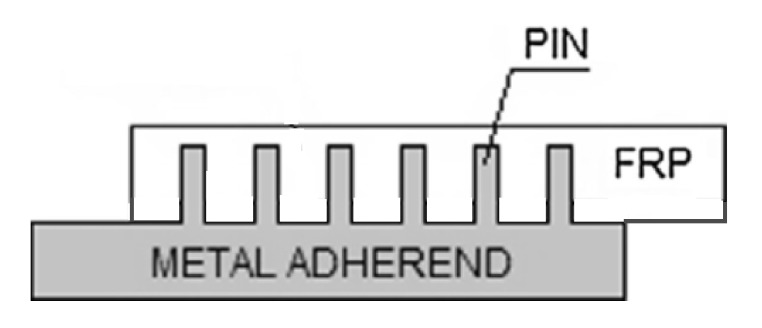
Schematic of an advanced hybrid single lap pin joint (section view). Pin joints combine adhesive bonding with an interlocking array of mechanical reinforcement. [62].

Another method of creating joints between the fibre-reinforced plastic composites and metal parts involves producing metal part with an array of pins protruding vertically from it. Once the array of pins has been prepared, it is necessary to integrate it with the composite material [62]. During the joint manufacturing the composite plies are placed on the top of the metal, so that their fibres are arranged around the pins and the pins penetrate the fabric layers throughout the thickness with little fibre damage leading to mechanical interlocking of the laminate with the metal structure [62,63,64] (Figure 18). This allows to achieve a joint without drilling holes in cured composite and without destroying the fibres [64]. Then the composite is cured. The cure methods suitable for curing pin joints include VARTM [62,63,64] and prepreg technology [62,65,66].

A number of techniques have been used to produce pins on the surface of metallic components for the purpose of hybrid joining [62]. Those techniques can be broadly categorised as surface restructuring or additive manufacturing (AM) [62]. A surface restructuring method by an electron beam called Comeld was developed by The Welding Institute [67]. The main drawbacks of the surface restructuring approach are limited control of pin geometry, excessive damage to the surface caused by the restructuring process and large costs associated with using an electron beam to drive the material across the surface [62]. Despite those drawbacks this method was used to produce pin joints of titanium and carbon-epoxy composite [67,68]. 

The main advantage of the pin joints is that the joining process does not require manufacturing holes in cured laminate, so problems like delaminations and stress concentrations are mitigated. The metal pins cause very slight disruption of the reinforcing fibres, which may contribute to the good performance of the joint. On the other hand, the disadvantages of the pin joints are following:

- The process of manufacturing the pin arrays on the metal substrate is complicated and, therefore, cost and time consuming.
- Once the joint is manufactured, it is impossible to disassembly it without destroying it.
- Galvanic corrosion may occur in this joining method if a substrate made of aluminium alloys or steel are used along with CFRP composite [22,23].

**Figure 19:**
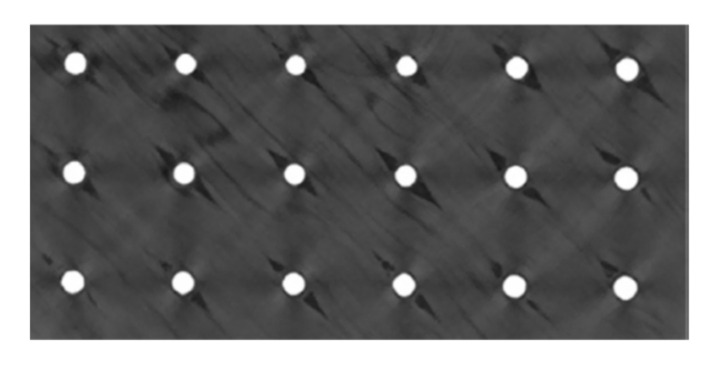
CT picture showing resin rich zones and fibre misalignment around pins [66].

Although the pin joining technique does not cause such strong destruction of the composite fibres as, for instance, hole drilling, it causes certain local disruption of the composite structure such as localised fibre waviness, broken fibres and a resin-rich zones [69]. Figure 19 shows a picture obtained by computer tomography (CT) of UD composite structure around the pins [66].

Composite–metal pin joints typically exhibit very low interface strength due to poor pin-composite bonding, which significantly affects joint performance [69]. The mismatch in thermal expansion coefficient (CTE) between the pins and composite leads to significant debonding at the pin-composite interface during curing in elevated temperature [70,71]. The ultrasonic inspection may be used to evaluate the general quality and detect failure of the pin joints [72].

**Figure 20:**
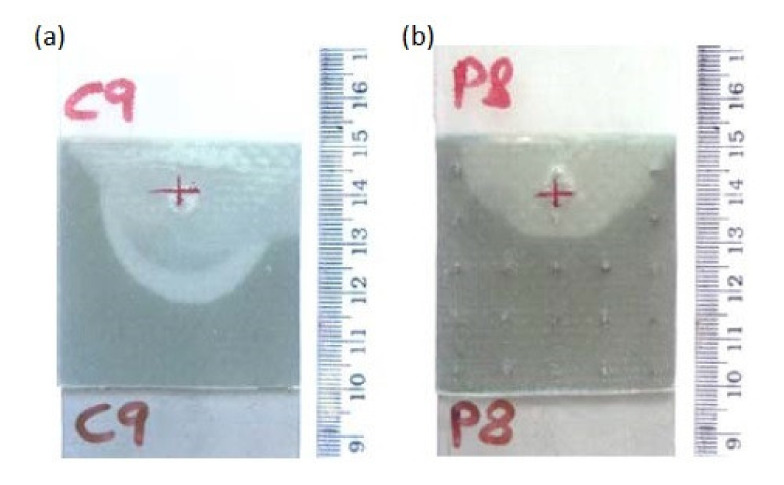
Images showing the level of disbonding for (**a**) control and (**b**) pinned joint after being subjected to a 13 J and 14 J impact respectively [62].

Parks et al. investigated the strength of single lap shear pin joints of titanium alloys and CFRP composite. The adherends were joined by 6 × 6 array of cylindrical pins protruding from the metal adherent in the area of 25.4 × 25.4 mm overlap [72]. The limit load was 21% higher and the ultimate load was 650% higher than the ultimate load of a comparable co-cured unpinned joint [72]. Graham et al. investigated pin joints of glass/epoxy fabric composite and stainless-steel substrate with square 8 × 8 pin arrays and compared them to control co-cured specimens without pins [62]. Single and double lap joints were subjected to fatigue loading. A 25-gsm glass fibre veil was included at the metallic interface of the prepreg joints in order to graduate the coefficient of thermal expansion (CTE) between the metallic and carbon fibre reinforced polymer adherends [62]. In both pin and control specimens the fatigue damage initiated at the ends of the overlap [62]. Damage within the control specimens progressed at an increasing rate following crack initiation. Damage in the hybrid pin joints initiated at a lower number of cycles, but the rate of damage growth reduced considerably as the crack front reached each row of reinforcing pins [62]. This is likely to be the result of enhanced load transfer through the pins as the bondline is damaged and the more highly stressed ‘joint edge’ effectively moves closer to the pins. This conclusion is supported by the observation that after a period of arrested crack growth, the respective row of pins experiences fatigue failure and the crack front begins to advance as the adhesive is reloaded [62]. Graham et al. also tested the strength and impact resistance of pin-joined 3-mm thick stainless steel and 2.5-mm thick glass fabric–epoxy composite [62,63]. Pin spacing was designed to locate at gaps in the weave architecture of the fabric to minimise fibre disruption. The experimental investigation was conducted using single lap joint specimens. These tests showed increases in strength and impact energy absorption (compared with control specimens—co-cured without pins), in the range of 70–100% and 300–800%, respectively [62,63]. The impact energy required to generate visible damage was slightly higher for hybrid joints while the extent of disbonding at higher impact energies was reduced significantly [62,63]. Two specimens subjected to similar impact loading, one control and one pin joint, are shown in Figure 20. It is apparent that, in the case of the pin joined specimen, the crack front has been arrested in a smooth curve bound by a number of pins [62,63].

It was also discovered that the pins not only decrease the area damaged by the impact, but also prevent the loss of the residual strength and the propagation of the damaged area. The improvement in mechanical performance results from the load distribution across the joint. As long as the reinforcing pins bridge the two substrates, they are capable of transmitting load and, therefore, reduce the stress intensity at the crack front [62,63]. The joint ultimately fails when the pins fracture at the base or are pulled out from the composite [62,63]. 

**Figure 21:**
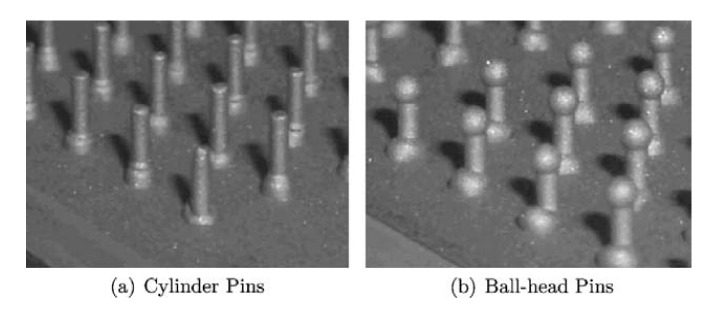
Two shapes of metal pins (**a**) cylinder and (**b**) ball-headed [64].

Uscnik et al. conducted tests of pin joints made of stainless steel and carbon reinforced thermoplastic composite with two kinds of pins manufactured on the surface of the steel substrates: cylinder and ball-headed (Figure 21) [64].

**Figure 22:**
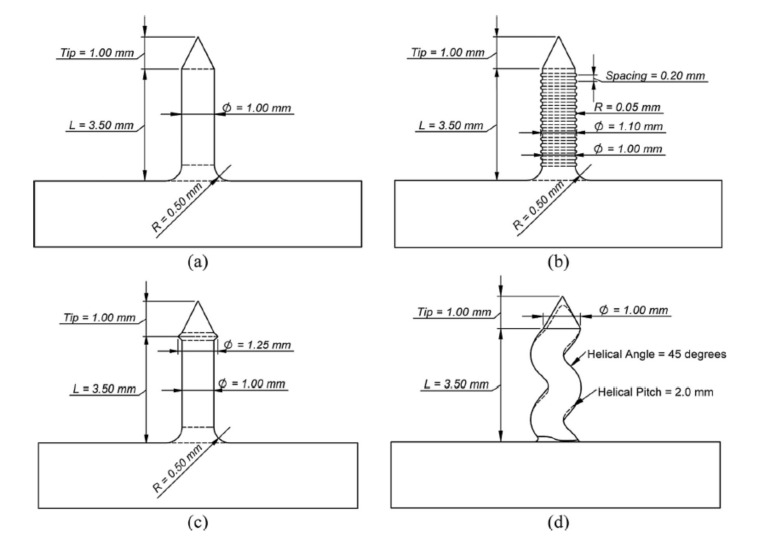
Single pin goemetries: (**a**) cylindrical, (**b**) grooved, (**c**) pyramid and (**d**) helical [65].

Double lap shear specimens were tested in tension. The specimens with cylinder and ball-headed pins exhibited different modes of failure. The cylindrical pins were pulled of the composite material, whereas the ball-headed pins were fractured as the bolt failed. It resulted in higher tensile strength of the joints with the ball-headed pins (150 N) than in the case of the cylindrical pin joints (110 N) [64]. The performance of titanium and carbon/epoxy prepreg tape composite pin joints with different pin geometries was also investigated by Nguyen et al. [65]. Four different shapes of pins (Figure 22) were tested on single pin specimens in pull-out test mode. The highest strength, higher by approximately 20% than for cylindrical pins was achieved for pyramid and helical pins. Lower force increase (by 14%) was obtained for the grooved pins. The mode of failure for cylindrical and grooved pins was pull-out, whereas for pyramid and helical pins, composite failure occurred. This indicates that the use of geometry features increases the pin joint strength, so that the composite strength becomes the limiting factor for joint behaviour in the case of those pin types [65].

**Figure 23:**
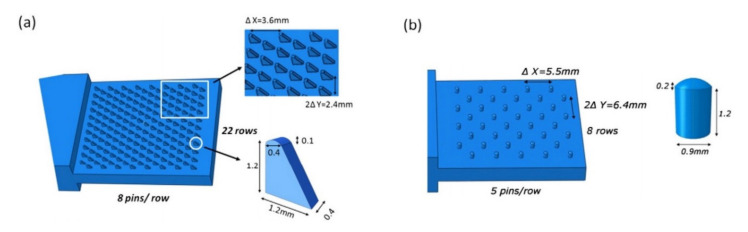
Profiles of different pin shapes (**a**) wedged-profiled and (**b**) cylindrical [68].

In the same work multi-pinned double cantilever beam (DCB) specimens were tested [65]. The cylindrical pins were manufactured with inclination angles 0° or 20° from the vertical direction (inclined along or against the crack growth direction). The results have shown that the influence of the pin inclination angle on the increase of the maximum load and fracture toughness in DCB specimens is negligible or may even deteriorate the performance of the specimens in the case of the pins inclined along the crack direction [65]. Experimental work made by other researchers [73,74] revealed significant changes of failure mode and energy absorption for end notched flexure (ENF) specimens under mode II loading when the inclination angle of pins was changed. In those specimens, changing the pin alignment meant that the axial component of the pin load was a compressive load for the case where the pin angle is against the shear load direction [73,74]. Tu et al. investigated the influence of the pin inclination in a lap shear metal-composite pin joint by the means of FEM optimisation [75]. It was concluded that some inclination angles may reduce the stress concentrations and thus improve the joint strength. The optimum angle for the pins is 20° to 30° towards the metal end of the joint [75]. Nguyen et al. also conducted another research in which the performance of titanium and carbon/epoxy prepreg tape composite pin joints were investigated taking into account the different sizes of cylindrical pins [69]. Multi-pinned double cantilever beam (DCB) specimens were tested with pins with different aspect ratios of 3.3 (D = 0.5, L = 2) and 6.5 (D = 0.5, L = 4). The DCB specimen with longer pins showed higher opening load and lower fracture toughness. Two aspects of pin joints of carbon fibre reinforced composite and titanium substrates were investigated by Parkes et al. [66]: the diameter of the pin root and the surface treatment of the titanium substrate. The two types of pins were manufactured: with conical head and with different root diameters, and one was 17% larger than the other. Some specimens were left with raw surface and the others were nano-structured with laser treatment. The specimens were subjected to single lap shear tensile tests. The increase of the pin root resulted in approximately 30% increase of the ultimate strength, whereas, the laser treatment had virtually no effect on the strength. These results are not surprising taking into account that the specimens failed by the fracture of the pins, so the pin root diameter had to have an influence on the ultimate strength. It can be also concluded that in the pin joints the surface treatment is unnecessary if the geometry of the pins is designed so that the joint fails by the fracture of the pins. Wang et al. tested a novel shape of the pins protruding from the metal substrate in the pin joints (Figure 23) [67,68]. The pins had wedged-profiled shape (Figure 23a). The joints between a titanium alloy and a carbon fibre reinforced polymer (CFRP) were made. The specimens were tested in double-lap shear tests. It was shown that the failure process consists of two main stages: the first stage involves the separation of the interfaces (debonding), the second stage consisted in the failure of the composite involving delamination, fibre fracture and some inter-ply matrix cracking [67]. In the second work, the results were compared to the results of joints with cylindrical pins (Figure 23b) [68].

The cylindrical-pinned joints failed by the pulling out of the pins from the CFRP component [68]. This resulted in lower failure strength of the specimens (30 kN) compared to the strength of the wedge-pinned joints (73 kN) [68]. However, the lower strength of the cylindrical-pinned joints may be also influenced by the lower density of the pins (Figure 23). Xiong et al. investigated the effect of composite orientation on the mechanical properties of the pin joints [76]. Carbon fabric reinforced epoxy composite was joined with titanium alloy component with protruding wedge-like protrusions similar as in the previous works [67,68]. The thickness of the composite adherend was kept constant and the volume content of ±45° ply was increased from 11.1% to 88.9% (the rest of the plies had orientation 0°). The specimens were tested in single lap shear experiments. With the increase of the ±45° plies content the failure mechanism changed from the composite matrix crushing to the combination of the bending, the fracture of pins and composite compression [76]. With optimum composite orientation (55.6% of 45° plies), the joints damage initiation load was increased by 24.84% and the joint ultimate failure load was increased by 134.5% compared to the reference specimens without protrusions [76]. 

General characteristics of pin joining are presented in Table 6.

## 6. Loop Joining

**Figure 24:**
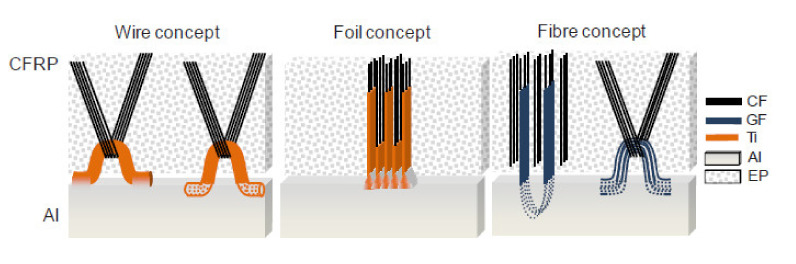
Concepts of joining CFRP to aluminium by loops, foils and fibres [77].

Research group “Schwarz–Silber” funded by German Research Foundation proposed a set of novel ideas for joining carbon fibre reinforced composites with aluminium alloys: wire, foil and fibre concepts [22,77,78] (Figure 24). Since the majority of the presented techniques include attaching loops to the aluminium alloy substrate, this group of techniques is referred to as ‘loop joining’ in the scope of the present work.

All the joining concepts are focused on separating carbon fibres from aluminium alloy in order to avoid galvanic corrosion, which appears inevitably when those two materials are in contact [22,23] and may lead to increased corrosive degradation on the aluminium [79] followed by a catastrophic failure of carbon/aluminium joints. The corrosion is caused by an electrochemical potential difference between carbon fibres and aluminium alloys [22] and may be avoided by connecting carbon fibres to the aluminium alloy substrate by transitional elements made of titanium, which reduce the electrochemical potential difference by about two-thirds in comparison to the combination of carbon fibre and aluminium and may be even decreased further by surface treatment of titanium [22], or even non-conductive materials such as glass and boron fibres [77]. The wire concept consists in connecting rows of titanium wire loops to the aluminium substrate by welding or casting [77]. The bundles of carbon fibres are then threaded through those loops. In the foil concept the loops are replaced by titanium foils, which are also connected to the aluminium substrate. The carbon fibres are inserted between the foils and the joint is held together by friction force [80]. In the fibre concept the titanium is replaced by glass or boron fibres [77]. All the joints are then embedded in polymeric resin. The presented concepts have some advantages:

- The transition elements insulating carbon fibres from aluminium alloy decrease or eliminate the possibility of galvanic corrosion [22,77].
- The continuity of carbon fibres is not disrupted in any way [77].

On the other hand, their disadvantages are following:

- Joining the transition elements (titanium, glass, or boron fibre) to aluminium substrate is tedious, time consuming and the joint often constitutes the weakest point of the structure [22,78,80,81].
- Glass and boron fibres have often lower strength than carbon fibres. This means that the ultimate strength of carbon reinforced composite structure is reduced to the strength of those fibres if they are used as the insulating elements.
- The additional elements in the joints increase the mass of the joint.

**Figure 25:**
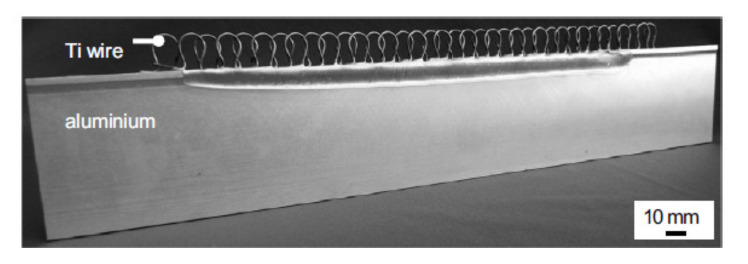
Aluminium structure with attached titanium wire loops [81].

Schumacher et al. investigated the joining of aluminium substrate and carbon fibres by titanium loops [78]. The loops were joined with the aluminium sheets by laser beam welding, during which the aluminium melts and wets the titanium wire, which stays in solid state [78]. The specimens with three or five loops were manufactured and a roving bundle was put through each loop and preloaded by a tension force. Subsequently, the rovings as well as the titanium transition structure were impregnated under vacuum by resin [78]. The specimens were subjected to tensile tests. The failure strengths were 3000 N/25 mm and 8000 N/43 mm respectively. However, all the specimens failed by the fracture of the titanium wire, which makes the results unsuitable for thorough evaluation of the joint strength. Moller et al. also used titanium loops joined to aluminium alloy structure by heat conduction laser beam welding process in order to join it with carbon fibre reinforced composite [81]. Only the aluminium structure was molten in order to create a bond between the aluminium and the titanium wire [81]. The titanium wire with a diameter of 0.8 mm was cold-formed to obtain a 2-dimensional loop structure with a principal radius of 2.5 mm. During processing, the laser beam was travelling along the aluminium edge, thus creating a molten aluminium pool. The aluminium wetted the titanium wire structure without melting it [81]. The resulting aluminium structure with titanium loops is presented in Figure 25 [81]. 

**Figure 26:**
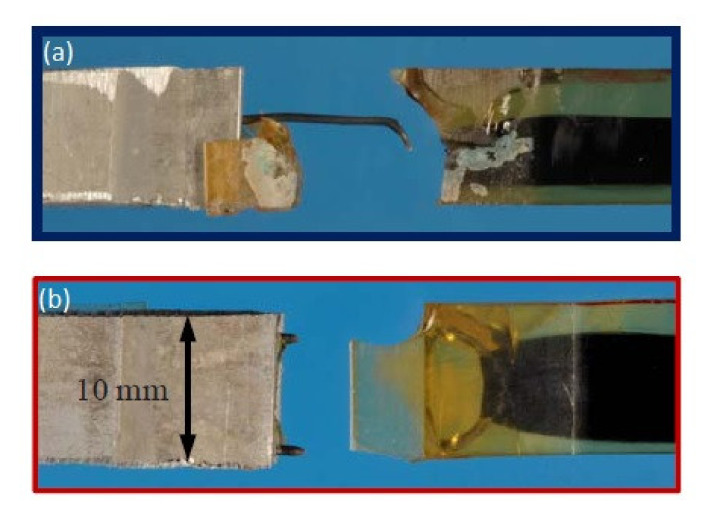
Failure behaviour of diffusion bonded Al-Ti-CFRP joint, in which Al-Ti joint was prepared at the process temperature (**a**) 480 °C and (**b**) 540 °C [22].

Carbon fibres were threaded through the loops and the structure was impregnated with polymeric resin in order to create CFRP composite. Tensile tests of such specimens were performed. The fracture started with delaminations of the matrix at the interfacial zone to the aluminium structure. The complete failure of the specimens occurred by subsequent failure of the titanium wires close to the aluminium weld. Only in some cases, the fracture path was through the aluminium substrate [81]. Taking into account that the metal joint failed before the composite, it is very difficult to assess the reliability of such an approach to composite-metal joining. Without increasing strength of the metal-metal joint, the full strength of the composite-material joint cannot be utilised. In the work by Schimanski et al. joints made of titanium wire loops connected with aluminium structure with carbon fibre bundles threaded through them and impregnated by polymeric resin were also investigated [22]. The investigation was focused on the manufacturing of a titanium–aluminium connection. The process used to join those two components was a combination of diffusion bonding and hot pressing [22]. The aluminium wire was formed into omega-shaped loops and placed in a 4 mm notch in the aluminium substrate. The whole sample was placed in a vacuum furnace between two plain plungers. To reinforce the contact between the titanium loops and the aluminium sheet, steel foils were placed at the lower and upper side of the aluminium sheet on the notch [22]. Two process temperatures were used: 480 °C and 540 °C. The mechanical properties of the joints with carbon roving threaded through the loops and embedded in polymeric resin were determined by tensile test. The specimens joined in 480 °C withstood approx. 300 N and the specimens joined in 540 °C, approx. 500 N [22]. The specimens joined in 480 °C failed by disconnection of the titanium wire from the aluminium substrate after cracking of the matrix (Figure 26a). The specimens joined in 540 °C failed by fracture of the wire in the vicinity of the substrate (Figure 26b).

**Figure 27:**
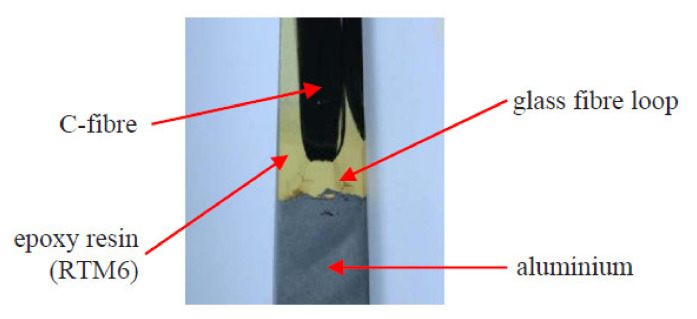
Connection of CFRP to aluminium alloy by loop made of glass fibres [82].

Clausen et al. used glass fibre loops to join carbon fibres to an aluminium alloy substrate [82]. In the investigated approach glass fibre loops were integrated with an aluminium alloy part, carbon fibre bundles were threaded through the loops, and the joint was impregnated by epoxy resin (Figure 27) [82].

**Figure 28:**
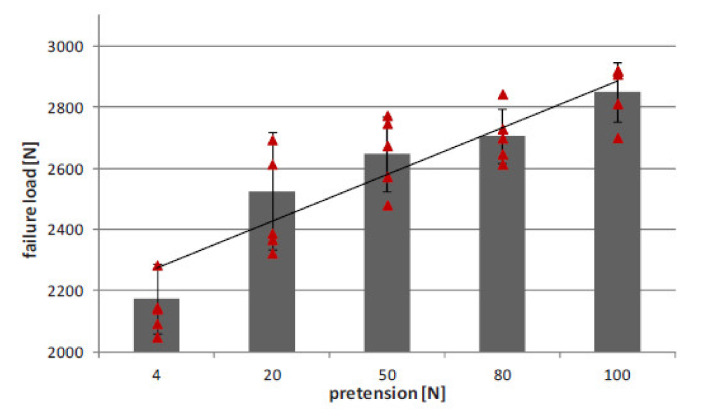
Failure load of Al-glass fibres-carbon fibres specimens embedded in epoxy matrix related to pretension force [77].

In the scope of the work, however, no tensile tests were performed in order to measure the strength of the joint. An assumption that the glass fibre loop joint is by 50% lighter than a bolted joint in aluminium/carbon fibre reinforced structure was made [82], however, no reasoning behind it was presented. Lang et al. investigated loops made of different fibres (boron, S-glass, and E-glass) which could be used for joining carbon fibre reinforced and aluminium structures [77]. Firstly, ‘dry’ specimens of selected fibres looped with different carbon fibres are tested for tensile failure strength. The pair of fibres for which the highest strength was achieved, S-glass fibre and HTS 24k carbon fibre, was selected for further tensile tests, in which the specimens were embedded in epoxy matrix. Before embedding the fibres in the resin, the carbon fibre loops were pre-tensioned with different forces (4, 20, 50, 80 and 100 N) [77]. The tensile strength increases steadily with the increasing pretension load from less than 2200 N for 4 N of pretension to over 2800 N for 100 N of pretension (Figure 28) [77].

The first fibre failures were predominantly located in carbon fibres [77]. This indicates the importance of using for the loop materials with a strength similar to or higher than the strength of joined carbon fibres. Otherwise, the loop fails first, deteriorating the performance of the carbon fibre reinforced structure.

**Figure 29:**
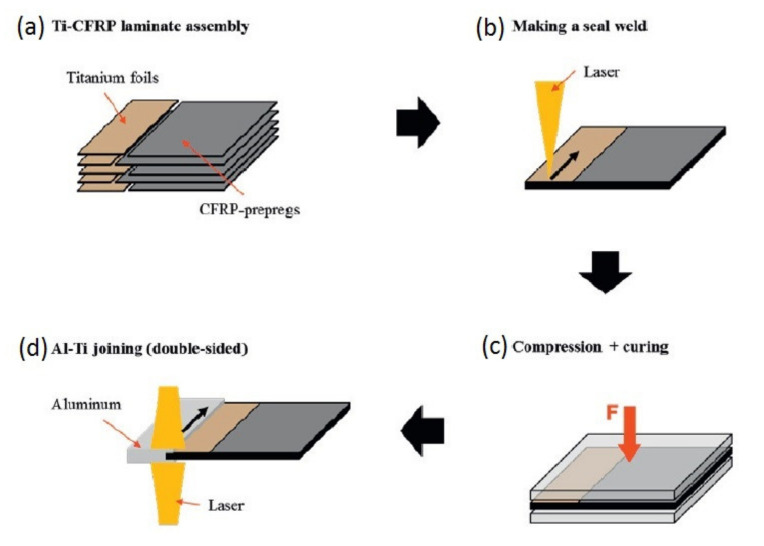
Procedure of the foil concept of joining CFRP to aluminium structures by laser beam: (**a**) Ti-CFRP laminate assembly, (**b**) making a seal weld, (**c**) compression and curing, (**d**) Al-Ti joining (double-sided) [80].

Woizeschke and Schumacher investigated the joining of aluminium alloy and carbon fibres with the use of titanium foil concept [80]. A laser beam was used to melt aluminium alloy which wetted the titanium foil surface. The foils 0.6 mm thick are made of pure titanium. The joint manufacturing process is presented in Figure 29. Firstly, the titanium foils are stacked alternately with CFRP prepreg plies (Figure 29a). The pure titanium side of the joint is then sealed by the laser welding in order to insulate the carbon fibres (Figure 29b). The welded titanium is then placed in a 2 mm deep groove made in the aluminium alloy substrate and the assembly is again welded by a laser beam (Figure 29c). Finally, the joint is compressed and subjected to elevated temperature in order to cure CFRP prepreg (Figure 29d) [80].

The manufactured specimens were subjected to tensile tests. Three different failure modes occurred [80]. Three of five specimens fractured within the Al–Ti fusion zone, with the failure located at the front side of the titanium. One specimen failed at the Ti–CFRP interface by delamination and one at both Al–Ti and Ti–CFRP interfaces. However, failing of the Al–Ti interface at the front side of the titanium laminate has been detected in all specimens [80]. Hence, a modification of the Al–Ti joining zone would be necessary to make the entire specimen suitable for higher loads [80]. 

General characteristics of loop joining are presented in Table 7.

## 7. Additive Manufacturing

As mentioned in Section 5, additive manufacturing (AM) can be used to manufacture pins array for pin joints. AM techniques vary considerably, but the principle is the same, to ‘build up’ features by sequentially adding layers of material to a substrate [62]. AM techniques based on metal-powder processing allow reasonable control of pin geometry and do not generally cause excessive damage to the existing surface [62]. Two common types of metal powder processing are applicable in pins manufacturing: selective laser melting (SLM) and laser metal deposition (LMD) [62]. SLM utilises a metal powder bed, over which a laser spot is focused to selectively melt layers of the material [62]. The SLM technique was utilised by Nguyen et al. to print an array of pins from Ti-64 [65]. LMD works by blowing metal powder into the focal point of a high-power laser [62]. The LMD technique was used to form arrays of protruding pins on stainless steel substrates by Graham et al. [62]. For research purposes these techniques are in many aspects ideal, but they remain a costly option for industry [62]. Cold metal transfer (CMT) is a relatively modern AM technique that allows droplets of molten metal wire to be deposited onto a metal substrate in progressive layers. Uscnik et al. developed a CMT method which consisted in arc-welding of one end of a wire to the metal substrate and then tearing the wire by applying resistive heating and tensile force [64]. Thus, pieces of welded wire formed an array of pins, with expected shapes and dimensions, integrally attached to the metal [64]. It is generally possible to perform each of those surface restructuring or additive manufacturing processes on a range of metals including steel, aluminium, and titanium alloys [62].

**Figure 30:**
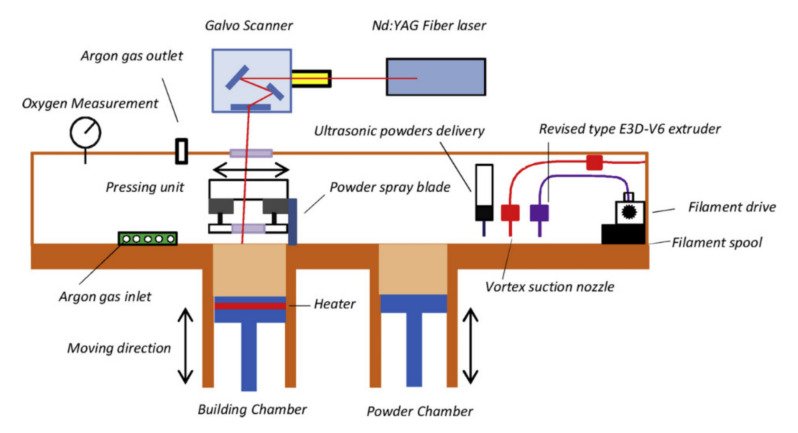
Printing system developed for additive manufacturing of joined metallic and polymer parts [83].

**Figure 31:**
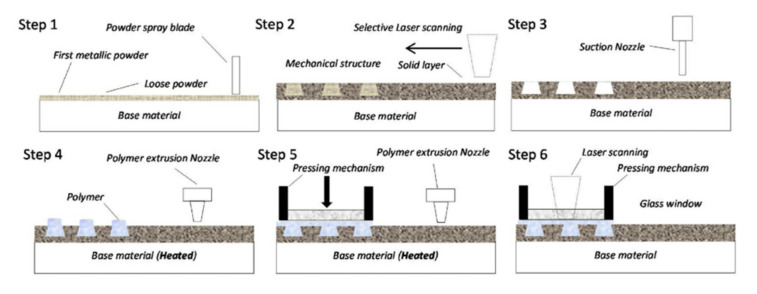
Sequence of additive manufacturing of joined metallic and polymer parts [83].

**Figure 32:**
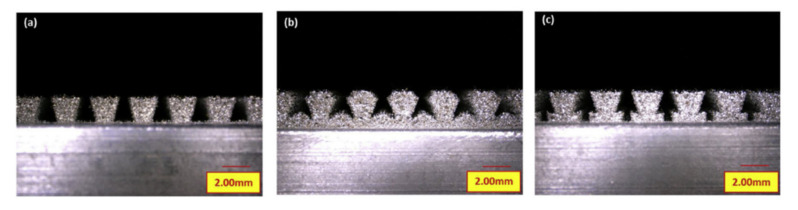
Various interlocking structures joining metallic and polymer parts [83] (**a**) “interlock contact”, (**b**) “root contact”, (**c**) “tree shaped contact”.

Additive manufacturing of both metallic and polymer composite is possible if short fibres are used to reinforce the polymer. Yuan-Hui Chueh et al. developed a method of integrated laser-based powder bed fusion and fused filament fabrication for three-dimensional printing of hybrid metal/polymer objects [83]. Laser-based powder bed fusion is used to manufacture array of metallic interlocking structures with predefined shapes on the metallic base. Then, polymer or reinforced polymer is extruded and pressed between these structures thus creating mechanical joint. In the course of polymer extrusion metallic base is heated facilitating penetration of the array with polymer. Pressing is performed with glass window with laser assist to improve the contact between interlocking structures and polymer. The printing system developed to manufacture joined metallic and polymer parts is presented in Figure 30 and Figure 31, which show the sequence of manufacturing. So far, pure metal polymer joints were manufactured and tested, but authors claim that composite made of polymer reinforced by short fibres can be also joined with metallic parts this way. Manufactured samples were investigated to reveal their strength. Two types of experiments were performed: shear tests and tensile tests. Samples were made of stainless steel, polyethene terephthalate (PET). Arrays of interlocking structures for shear tests had dimensions of 21 × 20mm whereas arrays for tensile tests had dimensions of 10 × 21 mm. Three shapes of interlocking structures were tested: “root contact”, “tree-shaped contact” and “interlocking contact” (Figure 32). In all cases, the shear strength of the joint appeared smaller than tensile strength. It did not exceed 18 MPa, whereas tensile strength exceeded 20 MPa for “root contact” and 22 MPa for two other shapes if interlocking structures. Moreover, tensile tests usually ended with fracture inside the polymer component whereas shear tests ended with fracture of polymer at the top if interlocking structure.

General characteristics of interlock joining with the application of integrated laser-based powder bed fusion and fused filament fabrication are presented in Table 8

An attempt to deposit melted metal (copper) at the top of polymer component was also presented in [83], but resulting joint strength appeared week. Laser energy small enough not to damage the polymer appeared not large enough to sinter the cooper powder well enough. Metal components can be manufactured after polymer ones if electroforming is applied instead [84]. It is possible because electroforming does not require high temperatures to deposit metals. Matsuzaki et al. investigated manufacturability of hybrid metal-polymer parts with application of fused filament fabrication and electroforming. They deposited one layer of polylactic acid (PLA) filament on top of the aluminium plate and then deposited copper in “moulds” created this way. This procedure was repeated several times allowing to build metal-polymer structure as high as 3.8 mm. To prevent coper deposition on other surfaces of an aluminium plate, it was covered by masking tape. In the areas where PLA and cooper were deposited, the aluminium plate was covered with conducting adhesive to prevent PLA from separating during plating process. Two types of pins were investigated “ordinary shaped” where metal pins were converging with height and “overhung shaped” where metal pins dimensions were growing with height. First type appeared more difficult to make because coper was deposited faster at edges of the metallic part then in its centre because higher concentration of the current around the edges. As a result, the thickness of the cooper at the edges was greater than the thickness of the PLA after each iteration leading to the collisions with printer head depositing PLA in the following step. This was not the case in the “overhung shaped” pins because printer head was not supposed to deposit PLA on the top of copper. It was enough to assume the ratio of the of metallic pin diameter increase to the PLA layer thickness to be smaller than ¾ to prevent copper layer against growing over the PLA layer. It was expanding radially instead. This phenomenon seems advantageous because it could allow for the production of effective interlocking structures if this method was applied to manufacture a metal–polymer connection. Unfortunately, authors investigated manufacturability only without any strength measurements, and therefore the strength of such a structure remains unknown. It seems reasonable to assume that PLA could be mixed with short fibres in this method, however it also seems like fibres would not reinforce the connection since they would not protrude from one layer to the following one inside the interlocking structure. Therefore, it seems like the strength of the connection would be similar for both pure PLA and PLA reinforced with short fibres. Moreover, it is not clear how strong the connection between the aluminium plate and the copper component of the interlocking structure is. However, other combinations of metals can be also applied.

General characteristics of pin joining with application of fused filament fabrication and electroforming are presented in Table 9. 

**Figure 33:**
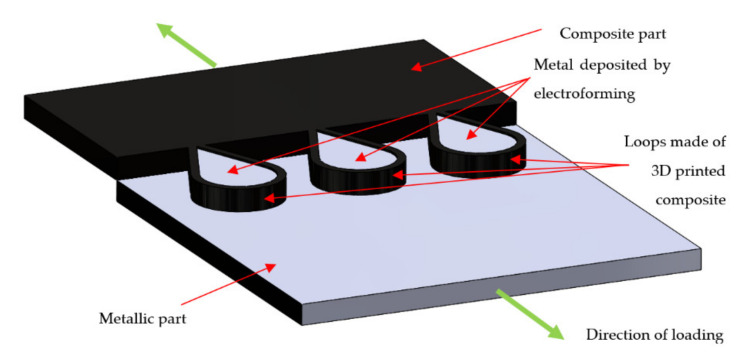
The concept of composite—metal joint made with application of 3D printing of continuous carbon fiber and electroforming.

It seems reasonable to combine electroforming [84] with loop joining [77,80] and 3D printing of continuous carbon fiber [85] or 3D printing of continuous-fiber composites by in-nozzle impregnation [86]. In both these methods polylactic acid reinforced by continuous carbon fibers was used to manufacture various parts including sandwich panels. To connect such a composite part with metallic part, the continuous carbon fiber composite part could be printed in the neighborhood of the metallic part with carbon loops extending from the composite part and touching the metallic part. Then the electroforming could be used to fill the composite loop with metal (Figure 33). The thickness of the loops can be increased by the following layers of the carbon fiber impregnated with polymer printed on the top of previous layers of the loop followed by the following electroforming sequences. At the end, the loops can be completely covered by the metal in the electroforming process. This idea would allow to create quite strong connection between composite part with metallic part with application of additive manufacturing only. Unfortunately, no publications on the application of such a method have been found. General characteristics of loop joining with application of 3D printing of continuous-fiber composites and electroforming are presented in Table 10.

## 8. Mechanical Joining of Metal and Composite Components Reinforced with Nanofibers

Carbon nanotubes are known as extremely strong objects with strength as high as 200 GPa [87]. Therefore, it seems reasonable to use them as a reinforcement of the polymer composite. Unfortunately, they are extremely slippery, so the adhesion between them and polymer matrix is very low [88,89]. As a result, reinforcing effect of carbon nanotubes is marginal for polymer matrix. That is why strength of the pure polymer is not much weaker than strength of the polymer reinforced by carbon nanotubes. Surface modification of nanotubes is envisaged as a solution of this problem [90], but it is possible only for multi-walled carbon nanotubes and decreases their strength since some carbon-carbon bonds have to be broken to attach additional particles. At the same time density of the reinforcement raises. Unfortunately progress in this technology have not been very rapid in recent years, so heavily loaded composite parts with carbon nanotubes as a main reinforcement have not became common yet. For example, Luo et al. [91] tried to modify multiwalled nanotubes by gliding arc plasma to reinforce polypropylene. As a result, they increased the strength of the material from 15.67 MPa for pure polypropylene and 31.32 MPa for composite consisting of polypropylene and untreated nanotubes up to 34.42 MPa for composite consisting of polypropylene and plasma treated nanotubes. Plasma treatment also improved dispersion of the nanotubes in the matrix. These results are quite impressive, but still far from the strength level required in the heavily loaded components. Therefore, dedicated methods of mechanical joining them with metallic parts have not been developed yet. On the other hand, carbon nanotubes and other nanofibers are quite promising as auxiliary reinforcement improving inter-laminar properties of composites reinforced by conventional fabrics [92,93,94,95,96,97,98]. For example, they could protect against delaminations in the case of self-piercing riveting or bolted joining. However, in this case, the type of main reinforcement determines the design of the metal–composite connection, so again, there is no point to develop a joining method dedicated particularly to metal with a nanofiber reinforced composite. On the other hand, every method applicable for composites reinforced by short fibres will also be applicable for composites reinforced by nanofibers.

Composite parts containing carbon nanotubes are currently much more useful in electronic applications [99] because of the interesting electric properties of nanotubes. They can exhibit both the conductor type of conductivity and semiconductor type of conductivity depending on their chirality [100]. Moreover, they can be filled with other chemical elements, including metals [101]. Enclosure of the metal atoms inside the carbon nanotube capsule which is dipped in the polymer matrix can be treated as a kind of mechanical joining of the metallic part with the nanotube reinforced polymer composite, but its applications are mainly in electronics and medicine, which exceeds the scope of this paper.

## 9. Summary

The techniques of the mechanical joining of composite and metal structures, namely self-piercing riveting, friction riveting, mechanical clinching, non-adhesive form-locked joining, pin joining, and loop joining, have been reviewed in the present work. All those methods constitute alternatives to traditional bolted joining. The concepts of those methods are so different that it is hard to compare them directly. However, the key features of bolted joining and those techniques are presented in Table 11. 

### 9.1. Disassembling

The first aspect of discussed joining methods is the possibility of disassembling and assembling of the joint without destroying it. Only bolted joining and non-adhesive form-locked joining methods have this advantage, however the only method which was designed purposefully to allow multiple assembly/disassembly of the joint is the form-locked joining. The bolted joints need additional protection (e.g., metal inserts [4]) in order to enable frequent assembly/disassembly without the destruction of the composite material. 

### 9.2. Complication and Damages Induced by Joining

Another category in which the joining methods can be evaluated are damages and flaws induced by the joining process. Significant mechanical and sometimes thermal damages are induced in bolted joining, self-piercing riveting, friction riveting and mechanical clinching, because the joining process requires cutting of the reinforcing fibres. However, in the case of the bolted joining there are some methods which can reduce the manufacturing damages [4]. The other three methods cause none or only slight flaws in the composite structure, because they are designed to join composite with metal in the stage of the composite part manufacturing. The complication level of the joining methods is another problem which may influence the choice of the joining method. Although the traditional bolted joining requires a separate step of hole manufacturing, this method is so common and well recognized that the complication level of the method can be assessed as low. The complication level increases if certain methods designed to reduce the damages induced by hole manufacturing process are employed. Self-piercing riveting, friction riveting and mechanical clinching are single-step operations and thus also have low complication level. On the other hand, form-locked, pin, and loop joining require manufacturing of certain geometrical features in order to assure the interlocking between the metal parts and the composite fibres. This causes a moderate or high complication level of the joint manufacturing process. A general conclusion can be drawn from the analysis of the process-induced damages and the complication level of the joining methods: the reduction of the process-induced damages requires the use of more sophisticated joining methods and thus increases the level of complication. 

### 9.3. Applicability to Thermoset and Thermoplastic Matrices

Another criterion used for the assessment of the joining techniques was their usefulness for joining thermoset matrix composites as well as thermoplastics. Bolted joining, self-piercing riveting, and mechanical clinching have been used for joining both thermoset and thermoplastic matrix composites. However, due to high strains caused in the composite by self-piercing riveting and mechanical clinching, it is discussible if those methods should be used for joining thermoset matrix composites. It seems that those methods can induce even more process-induced damages in those composites than bolted joining, but a separate investigation should be conducted in order to prove it. Friction riveting is used for thermoplastic composites only, because this method requires melting of the matrix. Non-adhesive form-locked joining and loop-joining have been used for joining only the thermoset composite matrix. The use of those methods for thermoplastic matrix composites, if possible, would require some modifications of the joining process.

### 9.4. Level of Development

The last but not least aspect of the discussed techniques is their level of development. The bolted joining is commonly used in all branches of composite industry. Self-piercing riveting and mechanical clinching are used in the automotive industry for creating composite-metal and metal-metal joints. Non-adhesive form-locked joints have been successfully used for creating fuselage-wing joints in gliders and motogliders. However, no proof of using friction riveting, pin, and loop joining in the industry has been found, though admittedly the friction riveting was considered as a method of creating joints in composite emergency bridges. Therefore, those methods require further and thorough investigation before employing them in commercial applications. The loop joining method in particular, in which the joining process of titanium loops to aluminium alloy structures seems faulty and unreliable, needs development and further tests if it is going to be used in industry.

### 9.5. Strength

The most crucial feature of a joint is its strength. Therefore, the comparison of the strengths requires a separate discussion. As the single lap shear testing is the most common method of testing joints in both composite and metal joints and it has been used to test the strength of the joints in many works cited in the present work, the comparison of SLS strengths seems to be the best mean to compare the mechanical performance of the described joining methods. Not all the methods though were investigated by SLS tests. The SLS testing is not applicable to non-adhesive form-locked and loop joining, because the first method is used to create joints too large to be the part of a standard SLS specimen, while the nature of the second method prevents the lap joining at all. Therefore, only bolted joining, self-piercing riveting, friction riveting, mechanical clinching, and pin joining were taken into account in Table 12 which compares the SLS strengths of the methods.

The maximum SLS strength results were selected for each method (Table 12). However, as they were collected from different works, the experimental conditions were not the same. All results, apart from the strength of the friction riveted joint, were obtained for CFRP composite. The use GFRP composite in the case of the friction riveted joint might have led to the underestimation of the joint strength if not for the mode of failure of the friction riveted specimen, which was rivet failure. However, the different modes of failures reported in the presented works constitute another problem in unbiased comparison of the strengths of joining methods. Moreover, the presented joints have different geometries. Admittedly, the diameters of bolted joint, SPR joint and friction riveted joint are similar, but the diameter of the joint used in the mechanical clinching is much larger and the geometry of the pin joint is so different from the other, that it explains the large dominance of the strength of this joint. In fact, the only two SLS strengths which can be compared properly are the strength of bolted joint and the strength of SPR joint, because the materials and the diameters of the joints are similar and they exhibit the same failure mode. Even though the thickness of the composite used for the bolted joining is slightly higher than for SPR joint (2 mm vs. 1.5 mm), the difference in strength is so high that it suggests the evident superiority of the bolted joint. In order to make a thorough and unbiased comparison of all the joining method strengths another experimental study should be conducted, in which selected methods should be tested in similar conditions. The comparison of specific strengths (strength related to the mass) of the joints would be also very useful.

### 9.6. Future Perspectives

In the future, we can expect further development of 3D printing technologies assuming that current problems with strength of composites reinforced with carbon nanotubes are solved. Full utilization of mechanical properties of nanotubes would allow for the creation of a new generation of composite materials. They would be characterised by mechanical properties never seen before. At the same time, they would be well suitable for 3D printing which should allow for rapid prototyping and manufacturing of parts with very complicated geometries and magnificent mechanical characteristics. Ability to create strong parts with complicated geometries will certainly facilitate mechanical joining with metallic parts if necessary.

In the meantime, pin joining and loop joining with application of 3D printing of continuous-fiber composites, electroforming, selective laser melting, and laser metal deposition could be investigated. Both these approaches seem to combine strength of composites reinforced by continuous fibers with rapid prototyping and/or manufacturing.

## 10. Conclusions

The present article contains a description of the mechanical methods suitable for joining fiber reinforced composite materials with metal elements with the exception of bolted joining, which is described in the accompanying paper [4]. A comparison of those methods with each other and bolted joining was made as well in order to simplify the choice of the optimal method for specific use. Crucial features of the methods such as the possibility of the joint disassembly without destroying it, the damages induced by the joining process, the complication level of the method, the possibility of using it for both thermoset and thermoplastic matrix composite, and the level of development were assessed. It was concluded that the reduction of the process-induced damages requires the use of more sophisticated joining methods and thus increases the level of the technique’s complication. An attempt to compare the SLS strength of the joining methods was also made. However, this attempt was not fully successful, because this method of testing is not applicable to some of the reviewed joining methods. Moreover, the SLS testing for the other methods was carried out for different materials and specimen geometries in each case, so no reliable general conclusion about the strength comparison can be drown. Such a comparison requires further investigation, preferably in a single research testing the strengths of different joining methods in similar conditions.

## Figures and Tables

**Table 1:** General characteristics of self-piercing riveting.

Possibility of Joint Disassembling	Process-Induced Damages and Flaws in Composite Material	Complication Level	Suitability for Thermoset/Thermoplastic Matrix Composites	Level of Technique Development
No	Significant mechanical damages	Low—a single step operation	Used for both, but due to high composite strains more advisable for thermoplastic	Successfully used in automotive industry

**Table 2:** Process windows for (**a**) AA6111-CFRP and (**b**) CFRP-AA6111 joints [43].

(a)						(b)					
Spindle Speed [rpm]	Feed Rate [mm/min]					Spindle Speed [rpm]	Feed Rate [mm/min]				
	60	120	270	420	600		60	120	270	420	600
3000	√	QI	QI	-	-	3000	√	√	QI	QI	-
6000	√	√	QI	QI	-	6000	√	√	√	QI	QI
9000	√	√	QI	QI	-	9000	√	√	√	√	QI

√ sound joint, QI quality issue, - not tested.

**Table 3:** General characteristics of friction riveting.

Possibility of Joint Disassembling	Process-Induced Damages and Flaws in Composite Material	Complication Level	Suitability for Thermoset/Thermoplastic Matrix Composites	Level of Technique Development
No	Significant mechanical and thermal damages	Low—a single step operation	Thermoplastic only	Specimen testing level. Considered as a method of joining of composite emergency bridges

**Table 4:** General characteristics of clinching.

Possibility of Joint Disassembling	Process-Induced Damages and Flaws in Composite Material	Complication Level	Suitability for Thermoset/Thermoplastic Matrix Composites	Level of Technique Development
No	Significant mechanical damages	Low—a single step operation	Used for both, but due to high composite strains more advisable for thermoplastic	Successfully used in automotive industry

**Table 5:** General characteristics of Non-adhesive form-locked joining.

Possibility of Joint Disassembling	Process-Induced Damages and Flaws in Composite Material	Complication Level	Suitability for Thermoset/Thermoplastic Matrix Composites	Level of Technique Development
Yes	No damages	Moderate complication during manufacturing process	Thermoset only	Successfully used in gliders and motogliders

**Table 6:** General characteristics of pin joining.

Possibility of Joint Disassembling	Process-Induced Damages and Flaws in Composite Material	Complication Level	Suitability for Thermoset/Thermoplastic Matrix Composites	Level of Technique Development
No	Low—flaws caused by fibre alignments	High—the pin manufacturing is tedious	Thermoset/thermoplastic	Specimen testing level

**Table 7:** General characteristics of loop joining.

Possibility of Joint Disassembling	Process-Induced Damages and Flaws in Composite Material	Complication Level	Suitability for Thermoset/Thermoplastic Matrix Composites	Level of Technique Development
No	No damages	High—joining loops and foils to aluminium substrate is tedious	Thermoset only	Specimen testing level. Unsuitable for use due to unreliability of the joining technique for the time being

**Table 8:** General characteristics of interlock joining with application of integrated laser-based powder bed fusion and fused filament fabrication.

Possibility of Joint Disassembling	Process-Induced Damages and Flaws in Composite Material	Complication Level	Suitability for Thermoset/Thermoplastic Matrix Composites	Level of Technique Development
No	No damages	High—dedicated 3D printer is required	Thermoplastic only	Specimen testing level.

**Table 9:** General characteristics of pin joining with application of fused filament fabrication and electroforming.

Possibility of Joint Disassembling	Process-Induced Damages and Flaws in Composite Material	Complication Level	Suitability for Thermoset/Thermoplastic Matrix Composites	Level of Technique Development
No	No damages	High—3D printer and equipment for electroforming required	Thermoplastic only	Specimen testing level.

**Table 10:** General characteristics of loop joining with application of 3D printing of continuous-fiber composites and electroforming.

Possibility of Joint Disassembling	Process-Induced Damages and Flaws in Composite Material	Complication Level	Suitability for Thermoset/Thermoplastic Matrix Composites	Level of Technique Development
No	No damages	High—dedicated 3D printer and equipment for electroforming required	Thermoplastic only	Mere idea

**Table 11:** Comparison of key features of mechanical joining methods suitable for joining composites to metals.

	Bolted Joining	Self-Piercing Riveting	Friction Riveting	Mechanical Clinching	Non-Adhesive Form-Locked Joining	Pin Joining	Loop Joining	Interlock Joining with Powder Bed Fusion and Fused Filament Fabrication	Pin joining with Application of Fused Filament Fabrication and Electroforming	Loop Joining with 3D Printing of Continuous Fibre and Electroforming
Possibility of joint disassembling	Yes	No	No	No	Yes	No	No	No	No	No
Process-induced damages and flaws in composite material	Significant, but possible to be reduced [4]	Significant mechanical damages	Significant mechanical and thermal damages	Significant mechanical damages	No damages	Low—flaws caused by fibre alignments	No damages	No damages	No damages	No damages
Complication level	Low or moderate—if manufacturing damages are to be reduced	Low—a single step operation	Low—a single step operation	Low—a single step operation	Moderate complication during manufacturing	High - the pin manufacturing is tedious	High-joining loops and foils to aluminium substrate is tedious	High-dedicated integrated 3D printer required	High-3D printer and equipment for electroforming required	High-dedicated 3D printer and equipment for electroforming required
Suitability for thermoset/thermoplastic matrix composites	Thermoset/thermoplastic	Used for both, but due to high composite strains more advisable for thermoplastic	Thermoplastic only	Used for both, but due to high composite strains more advisable for thermoplastic	Thermoset only	Thermoset/thermoplastic	Thermoset only	Thermoplastic only	Thermoplastic only	Thermoplastic only
Level of technique development	Commonly used in all branches of composite industry	Successfully used in automotive industry	Specimen testing level. Considered as a method of joining of composite emergency bridges	Successfully used in automotive industry	Successfully used in gliders and motogliders	Specimen testing level	Specimen testing level. Unsuitable for use due to unreliability of the joining technique for the time being	Specimen testing level	Specimen testing level	Mere idea

**Table 12:** Comparison of SLS strengths of joining methods.

	Bolted Joining [102]	Self-Piercing Riveting [19]	Friction Riveting [35]	Mechanical Clinching [48]	Pin Joining [66]
Maximum SLS strength	8 kN	3.7 kN	5 kN	3.4 kN	33 kN
Joined material	CFRP	CFRP	GFRP	CFRP	CFRP
Joint geometry	Diameter 4.8 mm	Diameter 4.7 mm	Diameter 5 mm	Diameter 8.2 mm	Aluminium pin array 6 × 6
Failure mode	Bearing	Bearing	Rivet failure	Pull-out	Pin failure

## References

[1] Shyha I.S., Soo S.L., Aspinwall D., Bradley S. (2010). Effect of laminate configuration and feed rate on cutting performance when drilling holes in carbon fibre reinforced plastic composites. J. Mater. Process. Technol..

[2] Lopez de Lacalle L.N., Lamikiz A., Campa F.J., Valdivielso A.F., Etxeberria I. (2009). Design and test of multi-tooth tool for CFRP milling. J. Compos. Mater..

[3] Lim T.S., Kim B.C., Lee D.G. (2006). Fatigue characteristics of the bolted joints for unidirectional composite laminates. Compos. Struct..

[4] Galińska A. (2020). Mechanical joining of fibre reinforced polymer composites to metals—A review. Part I: Bolted joining. Polymers.

[5] Cheng X., Wang S., Zhang J., Huang W., Cheng Y., Zhang J. (2017). Effect of damage on failure mode of multi-bolt composite joints using failure envelope method. Compos. Struct..

[6] Choi J.-I., Hashemina S.M., Chun H.-J., Park J.-C., Chang H.S. (2018). Failure load prediction of composite bolted joint with clamping force. Compos. Struct..

[7] Li R., Kelly D., Crosky A. (2002). Strength improvement by fibre steering around a pin loaded hole. Compos. Struct..

[8] Amancio-Filho S.T., dos Santos J.F. (2009). Joining of polymers and polymer-metal hybrid structures: Recent developments and trends. Polym. Eng. Sci..

[9] Kah P., Suoranta R., Martikainen J., Magnus C. (2014). Techniques for joining dissimilar materials: Metals and polymers. Rev. Adv. Mater. Sci..

[10] Pramanik A., Basak A.K., Dong Y., Sarker P.K., Uddin M.S., Littlefair G., Dixit S., Chattopadhyaya S. (2017). Joining of carbon fibre reinforced polymer (CFRP) composites and aluminium alloys—A review. Compos. Part. A Appl. Sci. Manuf..

[11] Dawei Z., Qi Z., Xiaoguang F., Shengdun Z. (2018). Review on joining process of carbon fiber-reinforced polymer and metal: Methods and joining process. Rare Metal Mater. Eng..

[12] Dawei Z., Qi Z., Xiaoguang F., Shengdun Z. (2019). Review on joining process of carbon fiber-reinforced polymer and metal: Applications and outlook. Rare Metal Mater. Eng..

[13] Job S., Worrall C., Kellar E., Vacogne C. (2020). Joining of Fibre-Reinforced Polymer Composites: A Good Practice Guide.

[14] Di Franco G., Fratini L., Pasta A., Ruisi V.F. (2013). On the self-piercing riveting of aluminium blanks and carbon fibre composite panels. Int. J. Mater. Form..

[15] Fratini L., Ruisi V.F. (2009). Self-piercing riveting for aluminium alloys-composites hybrid joints. Int. J. Adv. Manuf. Technol..

[16] Di Franco G., Fratini L., Pasta A. (2012). Influence of the distance between rivets in self-piercing riveting bonded joints made of carbon fiber panels and AA2024 blanks. Mater. Des..

[17] Settineri L., Atzeni E., Ippolito R. (2010). Self piercing riveting for metal-polymer joints. Int. J. Mater. Form..

[18] Di Franco G., Fratini L., Pasta A. (2012). Fatigue behaviour of self-piercing riveting of aluminium blanks and carbon fibre composite panels. J. Mater. Des. Appl..

[19] Di Franco G., Fratini L., Pasta A. (2013). Analysis of the mechanical performance of hybrid (SPR/bonded) single-lap joints between CFRP panels and aluminium blanks. Int. J. Adhes. Adhes..

[20] Gay A., Lefebvre F., Bergamo S., Valiorgue F., Chalandon P., Michel P., Bertrand P. (2016). Fatigue performance of a self-piercing rivet joint between aluminium and glass fiber reinforced thermoplastic composite. Int. J. Fatigue.

[21] Ueda M., Miyake S., Hasegawa H., Hirano Y. (2012). Instantaneous mechanical fastening of quasi-isotropic CFRP laminates by a self-piercing rivet. Compos. Struct..

[22] Schimanski K., von Hehl A., Zoch H.-W. (2013). Failure behaviour of diffusion bonded transition structures for integral FRP-aluminium compounds. Procedia Mater. Sci..

[23] Köhler J., Grove T., Maiß O., Denkena B. (2012). Residual stresses in milled titanium parts. Procedia CIRP.

[24] Starikov R., Schon J. (2001). Quasi-static behaviour of composite joints with countersunk composite and metal fasteners. Compos. Part. B Eng..

[25] Starikov R., Schon J. (2002). Fatigue resistance of composite joints with countersunk composite and metal fasteners. Int. J. Fatigue.

[26] Li D., Chrysanthou A., Patel I., Williams G. (2017). Self-piercing riveting—A review. Int. J. Adv. Manuf. Technol..

[27] Blacket S. The self pierce riveting process comes of age. Proceedings of the Materials in Welding and Joining Conference.

[28] Barnes T.A., Pashby I.R. (2000). Joining techniques for aluminium spaceframes used in automobiles: Part II—Adhesive bonding and mechanical fasteners. J. Mater. Process. Technol..

[29] Meschut G., Gude M., Augenthaler F., Geske V. (2014). Evaluation of damage to carbon-fibre composites induced by self-pierce riveting. Procedia CIRP.

[30] Gay A., Lefebvre F., Bergamo S., Valiorgue F., Chalandon P., Michel P., Bertrand P. (2015). Fatigue of aluminium/glass fiber reinforced polymer composite assembly joined by self-piercing riveting. Procedia Eng..

[31] Zhang J., Yang S. (2015). Self-piercing riveting of aluminum alloy and thermoplastic composite. J. Compos. Mater..

[32] Chishti M., Wang C.H., Thomson R.S., Orifici A.C. (2012). Experimental investigation of damage progression and strength of countersunk composite joints. Compos. Struct..

[33] Amancio-Filho S.T., Beyer M., dos Santos J.F. (2009). Method for connecting a metallic bolt to a plastic piece. U.S. Patent.

[34] Blaga L., Bancilā R., dos Santos J.F., Amancio-Filho S.T. (2013). Friction Riveting of glass-fibre-reinforced polyetherimide composite and titanium grade 2 hybrid joints. Mater. Des..

[35] Blaga L., dos Santos J.F., Bancila R., Amancio-Filho S.T. (2015). Friction Riveting (FricRiveting)as a new joining technique in GFRP lightweight bridge construction. Constr. Build. Mater..

[36] Altmeyer J., dos Santos J.F., Amancio-Filho S.T. (2014). Effect of the friction riveting process parameters on the joint formation and performance of Ti alloy/short-fibre reinforced polyether ether ketone joints. Mater. Des..

[37] Altmeyer J., Suhuddin U.F.H., dos Santos J.F., Amancio-Filho S.T. (2015). Microstructure and mechanical performance of metal-composite hybrid joints produced by FricRiveting. Compos. Part. B Eng..

[38] Amancio-Filho S.T. (2007). Friction riveting: Development and analysis of a new joining technique for polymer-metal multi-materials structures. Ph.D. Thesis.

[39] Gagliardi F., Conte R., Ciancio C., Simeoli G., Pagliarulo V., Ambrogio G., Russo P. (2018). Jouning of thermoplastic structures by Friction Riveting: A mechanical and a microstructural investigation on pure and glass reinforced polyamide sheets. Compos. Struct..

[40] Amancio-Filho S.T., Roeder J., Nunes S.P., dos Santos J.F., Beckmann F. (2008). Thermal degradation of polyetherimide joined by friction riveting (FricRiveting). Part I: Influence of rotation speed. Polym. Degrad. Stab..

[41] Borba N.Z., Blaga L., dos Santos J.F., Canto L.B., Amancio-Filho S.T. Friction riveting of pultruded thermoset glass fiber reinforced polyester composite and TI6AL4V hybrid joints. Proceedings of the Technical Conference and Exhibition ANTEC.

[42] Rodrigues C.F., Blaga L.A., dos Santos J.F., Canto L.B., Hage E., Amancio-Filho S.T. (2014). FricRiveting of aluminum 2024-T351 and polycarbonate: Temperature evolution, microstructure and mechanical performance. J. Mater. Process. Technol..

[43] Min J., Li Y., Li J., Carlson B.E., Lin J. (2015). Friction stir blind riveting of carbon fiber-reinforced polymer composite and aluminium alloy sheets. Int. J. Adv. Manuf. Technol..

[44] Gao D., Ersoy U., Stevenson R., Wang P.C. (2009). A new one-sided joining process for aluminum alloys: Friction stir blind riveting. ASME J. Manuf. Sci. Eng..

[45] Lathabai S., Tyagi V., Ritchie D., Kearney T., Finnin B. (2011). Friction stir blind riveting: A novel joining process for automotive light alloys. SAE Int. J. Mater. Manuf..

[46] Lee C.-J., Kim B.-M., Kang B.-S., Song W.-J., Ko D.-C. (2017). Improvement of joinability in a hole clinching process with aluminium alloy and carbon fiber reinforced plastic using a spring die. Compos. Struct..

[47] Lambiase F., Ko D.-C. (2016). Feasibility of mechanical clinching for joining aluminium AA6082-T6 and carbon fiber reinforced polymer sheets. Mater. Des..

[48] Lee S.-H., Lee C.-J., Lee K.-H., Lee J.-M., Kim B.-M., Ko D.-C. (2014). Influence of tool shape on hole clinching for carbon fiber-reinforced plastic and SPRC440. Adv. Mech. Eng..

[49] Lambiase F., Durante M., di Ilio A. (2016). Fast joining of aluminium sheets with glass fiber reinforced polymer (GFRP) by mechanical clinching. J. Mater. Process. Technol..

[50] Lambiase F., Paoletti A. (2018). Friction-assisted clinching of aluminum and CFRP sheets. J. Manuf. Process..

[51] Lambiase F., Ko D.-C. (2017). Two-steps clinching of aluminium and carbon fiber reinforced polymer sheets. Compos. Struct..

[52] Lee S.H., Lee C.J., Kim B.H., Ahn M.S., Kim B.M., Ko D.C. (2014). Effect of tool shape on hole clinching for CFRP with steel and aluminium alloy sheet. Key Eng. Mater..

[53] Gude M., Hufenbach W., Kupfer R., Freund A., Vogel C. (2015). Development of novel form-locked joints for textile reinforced thermoplastices and metallic components. J. Mater. Process. Technol..

[54] Lee C.J., Lee S.H., Lee J.M., Kim B.H., Kim B.M., Ko D.C. (2014). Design of hole clinching process for joining CFRP and aluminum alloy sheet. Int. J. Precis. Eng. Manuf..

[55] Lambiase F., di Ilio A. (2014). An experimental study on clinched joints realized with different dies. Thin Wall Struct..

[56] Abibe A.B., Amancio-Filho S.T., dos Santos J.F., Hage E. (2013). Mechanical and failure behaviour of hybrid polymer-metal staked joints. Mater. Des..

[57] Abibe A.B., Amancio-Filho S.T., dos Santos J.F., Hage E. (2011). Development and analysis of a new joining method for polymer-metal hybrid structures. J. Thermoplast. Compos. Mater..

[58] Buffa G., Baffari D., Campanella D., Fratini L. (2016). An innovative friction stir welding based technique to produce dissimilar light alloys to thermoplastic matrix composite joints. Procedia Manuf..

[59] Marjanowski J., Tomasiewicz J., Frączek W. (2017). The electric-powered motoglider AOS-71—The study of development. Aircr. Eng. Aerosp. Technol..

[60] Tomasiewicz J., Czarnocki P. (2016). Wing-to-fuselage attachment fitting for composite airframes—Experimental and finite element analysis. Compos. Theory Pract..

[61] Kelly G., Hallström S. (2004). Bearing strength of carbon fibre/epoxy laminates: Effects of bolt-hole clearance. Compos. Part. B Eng..

[62] Graham D.P., Rezai A., Baker D., Smith P.A., Watts J.F. (2014). The development and scalability of a high strength, damage tolerant, hybrid joining scheme for composite-metal structures. Compos. Part. A Appl. Sci. Manuf..

[63] Graham D.P., Rezai A., Baker D., Smith P.A., Watts J.F. A hybrid joining scheme for high strength multi-material joints. Proceedings of the 18th International Conference on Composite Materials.

[64] Uscnik S., Scheerer M., Zaremba S., Pahr D.H. (2010). Experimental investigation of a novel hybrid metal-composite joining technology. Compos. Part. A Appl. Sci. Manuf..

[65] Nguyen A.T.T., Amarasinghe C.K., Brandt M., Feih S., Orifici A.C. (2017). Loading, support and geometry effects for pin-reinforced hybrid metal-composite joints. Compos. Part. A Appl. Sci. Manuf..

[66] Parkes P.N., Butler R., Meyer J., de Oliveira A. (2014). Static strength of metal-composite joints with penetrative reinforcement. Compos. Struct..

[67] Wang X., Ahn J., Kaboglu C., Yu L., Blackman B.R.K. (2016). Characterisation of composite-titanium alloy hybrid joints using digital image correlation. Compos. Struct..

[68] Wang X., Ahn J., Lee J., Blackman B.R.K. (2016). Investigation on failure modes and mechanical properties of CFRP-Ti6Al4V hybrid joints with different interface patterns using digital image correlation. Mater. Des..

[69] Nguyen A.T.T., Brandt M., Feih S., Orifici A.C. (2016). Pin pull-out behaviour for hybrid metal-composite joints with integrated reinforcements. Compos. Struct..

[70] Mouritz A.P. (2012). Environmental durability of z-pinned carbon fibre–epoxy laminate exposed to water. Compos. Sci. Technol..

[71] Sweeting R.D., Thomson R.S. (2004). The effect of thermal mismatch on Z-pinned laminated composite structures. Compos. Struct..

[72] Parkes P.N., Butler R., Almond D.P. Growth of damage in additively manufactured metal-composite joints. Proceedings of the ECCM15—15th European Conference on Composite Materials.

[73] Cartié D.D.R., Cox B.N., Fleck N.A. (2004). Mechanisms of crack bridging by composite and metallic rods. Compos. Part. A Appl. Sci. Manuf..

[74] M’Membe B., Gannon S., Yasaee M., Hallett S.R., Partridge I.K. (2016). Mode II delamination resistance of composites reinforced with inclined Z-pins. Mater. Des..

[75] Tu W., Wen P.H., Hogg P.J., Guild F.J. (2011). Optimisation of the protrusion geometry in Comeld^TM^ joints. Compos. Sci. Technol..

[76] Xiong W., Blackman B., Dear J.P., Wang X. (2015). The effect of composite orientation on the mechanical properties on hybrid joints strengthened by surfi-sculpt. Compos. Struct..

[77] Lang A., Husemann L., Herrmann A.S. (2013). Influence of textile process parameter on joint strength for integral CFRP-aluminum transition structures. Procedia Mater. Sci..

[78] Schumacher J., Bomas H., Zoch H.-W. (2013). Failure behaviour of advanced seam structures for CFRP-aluminium connectors. Procedia Mater. Sci..

[79] Wang W.-X., Takao Y., Matsubara T. Galvanic corrosion-resistant carbon fiber metal laminates. Proceedings of the 16th international Conference on Composite Materials.

[80] Woizeschke P., Schumacher J. (2013). Failure behaviour of aluminium-titanium hybrid seams with a novel aluminium-CFRP joining concept. Phys. Procedia.

[81] Moller F., Thomy C., Vollertsen F., Schiebel P., Hoffmeister C., Herrmann A.S. (2010). Novel method for joining CFRP to aluminium. Phys. Procedia.

[82] Clausen J., Specht U., Busse M., Lang A., Sanders J. (2013). Integration of glass fibre structures in aluminium cast parts for CFRP aluminium transition structures. Procedia Mater. Sci..

[83] Chueh Y.-H., Wei C., Zhang X., Li L. (2020). Integrated laser-based powder bed fusion and fused filament fabrication for three-dimensional printing of hybrid metal/polymer objects. Addit. Manuf..

[84] Matsuzaki R., Kanatani T., Todoroki A. (2019). Multi-material additive manufacturing of polymers and metals using fused filament fabrication and electroforming. Addit. Manuf..

[85] Sugiyama K., Matsuzaki R., Ueda M., Todoroki A., Hirano Y. (2018). 3D printing of composite sandwich structures using continuous carbon fiber and fiber tension. Composites.

[86] Matsuzaki R., Ueda M., Namiki M., Jeong T.K., Asahara H., Horiguchi K., Nakamura T., Todoroki A., Hirano Y. (2016). Three-dimensional printing of continuous-fiber composites by in-nozzle impregnation. Sci. Rep..

[87] Thostenson E.T., Ren Z., Chou T.W. (2001). Advances in the science and technology of carbon nanotubes and their composites: A review. Compos. Sci. Technol..

[88] Lau K.-T., Shi S.-Q. (2002). Failure mechanisms of carbon nanotube/epoxy composites pretreated in different temperature environments. Carbon.

[89] Lau K.-T., Shi S.-Q., Cheng H.-M. (2003). Micro-mechanical properties and morphological observation on fracture surfaces of carbon nanotube composites pre-treated at different temperatures. Compos. Sci. Technol..

[90] Gojny F.H., Nastalczyk J., Roslaniec Z., Schulte K. (2003). Surface modified multi-walled carbon nanotubes in CNT/epoxy-composites. Chem. Phys. Lett..

[91] Luo Z., Cai X., Hong R.Y., Li J.H., Wei D.G., Luo G.H., Li H.Z. (2013). Surface Modification of Multiwalled Carbon Nanotubes via Gliding Arc Plasma for the Reinforcement of Polypropylene. J. Appl. Polym. Sci..

[92] Sihn S., Kim R.Y., Huh W., Lee K.H., Roy A.K. (2008). Improvement of damage resistance in laminated composites with electrospun nano-interlayers. Compos. Sci. Technol..

[93] Zhang J., Lin T., Wang X. (2010). Electrospun nanofibre toughened carbon/epoxy composites: Effects of polyetherketone cardo (PEK-C) nanofibre diameter and interlayer thickness. Compos. Sci. Technol..

[94] Kelkar A.D., Mohan R., Bolick R., Shendokar S. (2010). Effect of nanoparticles and nanofibers on Mode I fracture toughness of fiber glass reinforced polymeric matrix composites. Mater. Sci. Eng. B.

[95] Chen Q., Zhang L., Rahman A., Zhou Z., Wu X.F., Fong H. (2011). Hybrid multi-scale epoxy composite made of conventional carbon fiber fabrics with interlaminar regions containing electrospun carbon nanofiber mats. Compos. Part. A Appl. Sci. Manuf..

[96] Palazzetti R., Zucchelli A., Gualandi C., Focarete M.L., Donati L., Minak G., Ramakrishna S. (2012). Influence of electrospun Nylon 6,6 nanofibrous mats on the interlaminar properties of Gr–epoxy composite laminates. Compos. Struct..

[97] Razavi S.M.J., Neisiany R.E., Khorasani S.N., Ramakrishna S., Berto F. (2018). Effect of neat and reinforced polyacrylonitrile nanofibers incorporation on interlaminar fracture toughness of carbon/epoxy composite. Theor. Appl. Mech. Lett..

[98] Daelemans L., Cohades A., Meireman T., Beckx J., Spronk S., Kersemans M., De Baere I., Rahier H., Michaud V., Van Paepegem W. (2018). Electrospun nanofibrous interleaves for improved low velocity impact resistance of glass fibre reinforced composite laminates. Mater. Des..

[99] Pidcock G.C., in het Panhuis M. (2012). Extrusion Printing of Flexible Electrically Conducting Carbon Nanotube Networks. Adv. Funct. Mater..

[100] Liu J., Lu J., Lin X., Tang Y., Liu Y., Wang T., Zhu H. (2017). The electronic properties of chiral carbon nanotubes. Comput. Mater. Sci..

[101] Dorozhkin P.S., Tovstonog S.V., Golberg D., Zhan J., Ishikawa Y., Shiozawa M., Nakanishi H., Nakata K., Bando Y. (2005). A Liquid-Ga-Filled Carbon Nanotube: A Miniaturized Temperature Sensor and Electrical Switch. Small.

[102] Heimbs S., Schmeer S., Blaurock J., Steeger S. (2013). Static and dynamic failure behaviour of bolted joints in carbon fibre composites. Compos. Part. A Appl. Sci. Manuf..

